# Melinjo seed extract increases adiponectin multimerization in physiological and pathological conditions

**DOI:** 10.1038/s41598-020-61148-2

**Published:** 2020-03-09

**Authors:** Kentaro Oniki, Taisei Kawakami, Azusa Nakashima, Keishi Miyata, Takehisa Watanabe, Haruka Fujikawa, Ryunosuke Nakashima, Aoi Nasu, Yuka Eto, Noriki Takahashi, Hirofumi Nohara, Mary Ann Suico, Shunsuke Kotani, Yui Obata, Yuki Sakamoto, Yuri Seguchi, Junji Saruwatari, Tadashi Imafuku, Hiroshi Watanabe, Toru Maruyama, Hirofumi Kai, Tsuyoshi Shuto

**Affiliations:** 1 Division of Pharmacology and Therapeutics, Graduate School of Pharmaceutical Sciences, Kumamoto University, Kumamoto, Japan; 2 Department of Molecular Medicine, Graduate School of Pharmaceutical Sciences, Kumamoto University, Kumamoto, Japan; 3 Department of Molecular Genetics, Graduate School of Medical Sciences, Kumamoto University, Kumamoto, Japan; 4 Departments of Gastroenterology and Hepatology, Faculty of Life Sciences, Kumamoto University, Kumamoto, Japan; 5 Department of Organic Chemistry, Graduate School of Pharmaceutical Sciences, Kumamoto University, Kumamoto, Japan; 6 Department of Biopharmaceutics, Graduate School of Pharmaceutical Sciences, Kumamoto University, Kumamoto, Japan

**Keywords:** Obesity, Nutrition

## Abstract

Melinjo seed extract (MSE) contains large amounts of polyphenols, including dimers of trans-resveratrol (*e.g.* gnetin C, L, gnemonoside A, B and D), and has been shown to potentially improve obesity. However, there is no clinical evidence regarding the anti-obesity effects of MSE, and its mechanisms are also unclear. We investigated the hypothesis that MSE supplementation increases the adiponectin (APN) multimerization *via* the up-regulation of disulfide bond A oxidoreductase-like protein (DsbA-L) under either or both physiological and obese conditions. To investigate the effect of MSE on the physiological condition, 42 healthy young volunteers were enrolled in a randomized, double-blind placebo-controlled clinical trial for 14 days. The participants were randomly assigned to the MSE 150 mg/day, MSE 300 mg/day or placebo groups. Furthermore, in order to investigate the effect of MSE on APN levels under obese conditions, we administered MSE powder (500 or 1000 mg/kg/day) to control-diet- or high-fat-diet (HFD)-fed C57BL/6 mice for 4 weeks. All participants completed the clinical trial. The administration of MSE 300 mg/day was associated with an increase in the ratio of HMW/total APN in relation to the genes regulating APN multimerization, including *DsbA-L*. Furthermore, this effect of MSE was more pronounced in carriers of the *DsbA-L* rs191776 G/T or T/T genotype than in others. In addition, the administration of MSE to HFD mice suppressed their metabolic abnormalities (*i.e.* weight gain, increased blood glucose level and fat mass accumulation) and increased the levels of total and HMW APN in serum and the mRNA levels of *ADIPOQ* and *DsbA-L* in adipose tissue. The present study suggests that MSE may exert beneficial effects *via* APN multimerization in relation to the induction of *DsbA-L* under both physiological and obese conditions.

## Introduction

Melinjo (*Gnetum gnemon L*.) is a gymnosperm native of Indonesia, and its seeds have long been eaten by the people of Indonesia^1^. Melinjo seed extract (MSE) contains large amounts of polyphenols, including trans-resveratrol, gnetin C (dimer of trans-resveratrol), gnemonoside A (di-glucoside from gnetin C) and gnemonoside D (mono-glucoside from gnetin C), gnemonoside C (mono-glycoside from gnetin C) and gnetin L (derivative of gnetin C)^1^. Resveratrols, in particular trans-resveratrol, have been reported to have diverse health benefits, demonstrating utility for treating aging-related diseases, including cancer, cardiovascular disease, type 2 diabetes and neurodegenerative disease in animal studies^2–4^; however, its health benefits in humans are still controversial. One reason for this lack of certainty regarding its effects may be the rapid and presystemic metabolism of trans-resveratrol (*i.e.* low bioavailability)^3,4^. Since most of the resveratrol derived from MSE is gnetin C, which has a longer retention time in blood than trans-resveratrol (mean residence time: 36.3 h [gnetin C] and 6.6 h [trans-resveratrol], respectively)^5^, MSE may have more beneficial effects than trans-resveratrol. Moreover, since MSE contains several resveratrols^1^, the administration of MSE is also expected to have additional and/or diverse effects due to their combination. On the other hand, the fact that melinjo seeds are traditionally ingested as food in Indonesia, and the results of several clinical trials using MSE, indicate that MSE is safe for human administration^5,6^. MSE has been reported to have beneficial health effects for metabolic abnormalities (*e.g.* improvement of insulin resistance^7^, reduction in serum uric acid^6^ and inhibition of endothelial senescence^8^). However, the details of the health benefits of MSE as a functional food against metabolic abnormalities are unclear. To clarify the usefulness of MSE as a functional food for the prevention and suppression of metabolic abnormalities, the effects of MSE must be investigated under both physiological and pathological conditions.

Adiponectin (APN), one of the most abundant adipocytokines secreted from adipose tissues, has insulin-sensitizing, anti-inflammatory and cardioprotective properties^9–11^. The serum APN level is decreased in obese, insulin-resistant, type 2 diabetic rodents and humans^10,11^. APN circulates in three different multimer forms: trimers (low-molecular-weight [LMW]), hexamers (middle-molecular-weight [MMW]) and larger multimers (high-molecular-weight [HMW])^10,11^. Previous epidemiological evidence has shown that individuals with obesity, diabetes and/or coronary heart disease had decreased levels of APN, especially HMW APN^10–13^. Furthermore, the ratio of HMW to total APN is markedly correlated with the angiographic coronary atherosclerosis severity^14^. Therefore, the upregulation of endogenous APN multimerization may help prevent various metabolic diseases.

The APN multimerization process is positively regulated by disulfide bond A oxidoreductase-like protein (DsbA-L)^15^. DsbA-L is highly expressed in the endoplasmic reticulum (ER) and mitochondria^11^, and the mRNA level in adipose tissue was shown to correlate negatively with obesity/overweight in both mice and humans^15^. Previous *in vitro* and *in vivo* studies have shown that trans-resveratrol increased the APN multimerization *via* the activation of DsbA-L^16,17^; however, this effect in humans remains unclear. Furthermore, the effects of MSE administration on APN multimerization are also unclear.

There may be the individual difference in the effects of MSE administration on APN multimerization due to genetic polymorphisms in genes associated with APN multimerization. There is a common polymorphism of the *DsbA-L* gene at -1308 bp (rs1917760), which can influence the DsbA-L expression and/or activity^18^. In addition, there are common polymorphisms of the adiponectin gene (*ADIPOQ*) at +712 G > A (rs3774261) and -10066 G/A (rs182052), which can influence the APN protein level^19,20^.

The main objective of this study was to assess the usefulness of MSE as a functional food for the prevention and suppression of metabolic abnormalities by investigating the effects of MSE under both physiological and pathological conditions. To clarify the effects of MSE on APN multimerization under physiological conditions, we performed a double-blind, randomized placebo-controlled clinical study among healthy young subjects. In addition, we verified whether or not the effects of MSE on the APN levels were observed under obese conditions by administering MSE to control-diet- as well as high-fat-diet (HFD)-fed mice, while also taking into account MSE’s potential mechanism of action in the specific tissue.

## Materials and Methods

### Materials

MSE powder, tablets of MSE [75 mg], MSE [150 mg] and placebo were delivered in the same appearance and were supplied by the Yamada Bee Company, Inc. (Okayama, Japan). Melinjo seeds (*i.e.* the source of MSE tablets) were collected in Indonesia (Desa Bangkok, Kecamatan Gurah Kabupaten Kediri, Kediri, Jawa Timur). MSE powder was extracted from melinjo seeds using the method reported in a previous study^5^. The MSE powder was standardized to include minimum 20.0% resveratrol derivatives and contained 0.1% trans-Resveratrol, 1.7% Gnetin C, 20.0% Gnemonoside A, 3.8% Gnemonoside D and 9.0% dextrin. One MSE tablet [75 mg] contained 75 mg MSE powder, 75 mg dextrin and 80 mg cellulose. One MSE tablet [150 mg] contained 150 mg MSE powder, 80 mg cellulose. One placebo tablet contained 135 mg dextrin and 80 mg cellulose. The component analysis of the MSE powder was carried out using the methods reported in the previous study^5^ (Supplementary Fig. S1).

### Participants and clinical trial design

To determine the effect of MSE administration on APN multimerization under physiological conditions and to evaluate whether MSE administration has health benefits, even in healthy individuals, we analyzed the data in healthy subjects to avoid the confounding effects of other disease conditions. The clinical trial was conducted with 42 healthy young male Japanese volunteers in Kumamoto, Japan. The following inclusion criteria were used for recruitment: (1) men over 20 years of age, (2) having no history of smoking and (3) having no history of diseases that alter the metabolism and/or excretion of drugs (such as gastroenterological, cardiovascular, pulmonary or hepatic diseases). In addition, the following exclusion criteria were applied: (1) a history of allergy to any foods and drugs, (2) eating functional foods routinely involved in the metabolism of glucose and/or lipids, (3) drinking red wine routinely, (4) having taken part in another clinical trial within three months and (5) taking any internal medication within one week before the start of the clinical trial through the end of the clinical trial.

This prospective, randomized, parallel, double-blind placebo-controlled clinical trial was designed to assess the efficacy of MSE (150 or 300 mg/day) on increasing APN multimerization in healthy volunteers (Supplementary Fig. S2). The doses of MSE and the study period in the clinical trial were determined with reference to previous studies on the efficacy and safety of MSE and resveratrols^5,6,21,22^. MSE was reportedly well tolerated at a dose of 5,000 mg/day for 28 days with no serious adverse events in healthy volunteers^5^. A previous clinical trial showed that the oral administration of MSE 750 mg/day reduced the serum uric acid level and increased the high-density lipoprotein cholesterol level in healthy male volunteers^6^. However, there have been few papers on clinical trials that investigated the effectiveness of MSE, and the optimal dose of MSE in humans has not been determined. In previous clinical trials using resveratrol in healthy adults, the administration of 150 mg/day to 400 mg/day of resveratrol improved the metabolic profile (*e.g*. reduced blood glucose level and adipocyte size, ameliorated insulin sensitivity and protected against atherosclerosis)^22^. Most of the resveratrol derivatives in MSE are dimers of resveratrol (*i.e.* gnetin C and its glycosides) with a blood retention time approximately six-fold longer than that of resveratrol^5^. Therefore, in the present study, the doses of MSE were set at 150 mg/day (containing 30 mg resveratrol derivatives per day) and 300 mg/day (containing 60 mg resveratrol derivatives per day). The participants were randomly assigned to the MSE 150 mg/day (n = 14), MSE 300 mg/day (n = 14) or placebo (n = 14) groups *via* a double-blind procedure. Two tablets of MSE [75 mg], 2 tablets of MSE [150 mg] and 2 placebo tablets were administered to the subjects in the groups of MSE 150 mg/day, MSE 300 mg/day and placebo, respectively, every morning for 14 days. Participants were not allowed to take any medication or consume any beverages or foods containing resveratrol derivatives, grapefruit or alcohol from 48 h before the administration of test tablets until the end of the clinical trial. Compliance with test tablets was assessed using a pill count and self-reporting of missed doses during the study period. A whole-blood sample was collected from each participant on the day before taking test tablets (*i.e.* baseline) and on the 14th day after taking test tablets (day 14). The blood sampling time was set at 8:00 am. The biochemical examination as well as measurements of the APN levels and mRNA expression were performed using peripheral blood collected at baseline and day 14.

The number of clinical trial participants was determined in order to detect a 5% difference in the HMW to total APN ratio between baseline and day 14 with a power of 0.80 (a-level of 0.05).

The study was conducted according to the Declaration of Helsinki, and the protocol of our study was approved by the ethics committee of the Faculty of Life Sciences, Kumamoto University. All subjects provided their written informed consent prior to participation in the study. The clinical trial was registered at the UMIN Clinical Trials Registry (UMIN ID: UMIN000025643, https://upload.umin.ac.jp/cgi-open-bin/ctr_e/ctr_view.cgi?recptno = R000029504) on January 12, 2017.

### Mice and dietary treatment

C57BL/6 J mice were housed in a vivarium in accordance with the guidelines of the animal facility center of Kumamoto University. The light/dark cycle was set at 12 h, and the temperature was kept at a room temperature of 20 °C. To prepare diabetes model with HFD loading, an HFD purchased from Oriental Yeast Co., Ltd. (Tokyo, Japan) was used as previously described^23^. HFD was composed of 14% lard, 14% beef tallow, 25% casein, 20% sucrose, 15% cornstarch, 5% cellulose.

Five-week-old mice were randomly divided into 4 groups and given the following diet for 10 weeks: control diet for 10 weeks (n = 6); HFD for 10 weeks (n = 6); HFD for first 6 weeks and HFD supplemented with MSE powder 500 mg/kg/day for last 4 weeks (n = 6); and HFD for the first 6 weeks and HFD supplemented with MSE powder 1,000 mg/kg/day for the last 4 weeks (n = 6). HFD was orally administered to mice. Adipose tissue, muscle and liver samples were collected from mice that completed the diet. Since MSE powder has been reported to be well tolerated at an oral administration of 5,000 mg/day in humans^5^, the dose of 1,000 mg/kg/day for mice was calculated using a human equivalent dose (5,000 mg/60 kg/day in human)^24^.

All experiments on mice were approved by the Animal Welfare Committee of Kumamoto University and were conducted in adherence to institutional regulations and guidelines.

### Genotyping

In the human study, genomic DNA was extracted from whole blood using a DNA purification kit (FlexiGene DNA kit, Qiagen, Hilden, Germany). The genotypes of *DsbA-L* rs1917760 (-1308G > T), *ADIPOQ* rs3774261 ( + 712 G > A) and rs182052 (-10066 G/A) were determined using a real-time TaqMan allelic discrimination assay (Applied Biosystems, Waltham, MA, USA) according to the manufacturer’s protocol (assay Nos. C_7241_10, C_27479710_10 and C_2412785_10, respectively).

### Measurement of the mRNA expression

In the human study, total RNA in peripheral blood mononuclear cells (PBMCs) was extracted from whole-blood samples collected at the baseline and day 14 using the RNAiso Blood kit (Takara Bio Inc., Shiga, Japan) according to the manufacturer’s instructions. The integrity of the mRNA was checked on a 1% agarose gel and quantified at the 260/280 ratio. All mRNA samples were free of contaminating genomic DNA, so the mRNA was converted to cDNA using the Prime Scripts RT Reagent Kit with gDNA Eraser (Perfect Real Time) (Takara Bio Inc.). TaqMan quantitative polymerase chain reaction (PCR) was performed using a StepOnePlus Real-Time PCR system version 2.1 (Applied Biosystems) for *DsbA-L*, *forkhead box protein* (*Fox*) *O1*, *peroxisome proliferator-activated receptor* (*PPAR*) *γ*, *ER protein* (*ERp*)*-44*, *endoplasmic reticulum oxidoreductase 1-like protein* (*Ero1-L) α* and 5′*-adenosine monophosphate-activated protein kinase* (*AMPK*) (assay Nos. Hs01114170_m1, Hs00231106_m1, Hs01115513_m1, Hs00383195_m1, Hs00205880_m1 and Hs01562315_m1, respectively). Glyceraldehyde3-phosphate dehydrogenase (GAPDH; assay no. Hs99999905_m1) was used as an internal control. The expression was analyzed using the comparative 2^−ΔΔCt^ method^25^.

In the mouse study, murine tissues (adipose tissue, muscle and liver) was collected, and obtained samples were immersed in RNAlater solution® (Ambion, Austin, TX) and rotated overnight at 4 °C (except adipose tissue). Total RNA from murine tissues was isolated using RNAiso Plus (Takara Bio Inc.) according to the manufacturer’s instructions under homogenizing condition with a Wheaton homogenizer (Wheaton Industries, Millville, NJ) (for liver) or a Micro Smash MS-100 (4,000 rpm for 30 sec × 3 at 2 °C; TOMY SEIKO, Tokyo, Japan) with 1.0 g of zirconia bead (1.00 mm in diameter) and one stainless bead (4.8 mm in diameter; BioSpec Products, Bartlesville, OK, USA) (for adipose tissue and muscle). A real-time quantitative reverse transcription (RT)-PCR analysis of *DsbA-L*, *ADIPOQ*, *peroxisome proliferator-activated receptor gamma coactivator* (*PGC*) *1α*, *PPARα*, *sterol regulatory element-binding protein* (*SREBP*) *1c* and *GAPDH* was performed using SYBR® Premix Ex Taq™ II (Perfect Real Time) (Takara Bio Inc.) according to the manufacturer’s instructions. The relative quantity of *DsbA-L*, *ADIPOQ*, *PGC-1α*, *PPARα* and *SREBP1c* genes was normalized using either murine *GAPDH* gene as the internal controls and expressed as the relative quantity of target genes (fold induction). Primers used for real-time quantitative RT-PCR are shown in Supplementary Table S1.

### Measurement of APN protein levels

In both the human and mouse studies, the total and HMW APN levels in serum were determined by enzyme-linked immunosorbent assay (ELISA) kits (Human Total Adiponectin/Acrp30 and Human HMW Adiponectin/Acrp30 Quantikine ELISA Kits; R&D Systems, Minneapolis, MN, USA; or Adiponectin [HMW & Total] ELISA, Mouse; BioVendor Laboratory Medicine Inc., Brno, Czech Republic). The assays were performed according to the manufacturer’s protocol. In the mouse study, since APN levels are affected by fat mass, the obtained levels were divided by the fat mass.

### Measurement of oxidized albumin

In the human study, the redox state of albumin was measured as a systemic oxidative stress marker^26,27^ using serum samples with high-performance liquid chromatography (HPLC)^28^. Based on the HPLC profiles of HSA, the values of each of the albumin fractions (for human mercapto-albumin [HMA], human non-mercapto-albumin [HNA]1 and HNA2) were estimated by dividing the area of each fraction by the total area corresponding to albumin. A mixture of HNA1 and HNA2 was defined as oxidized albumin.

### Statistical analyses

The data are presented as the mean ± standard deviation, geometric mean (range) or frequency (%) of the subjects. Categorical variables were compared using Fisher’s exact test. A one-way analysis of variance (ANOVA) and Kruskal-Wallis test were used to compare the differences in the continuous parametric and nonparametric valuables, respectively. Dunnett’s procedure was used for multiple comparisons. In the human study, differences in variables between baseline and day 14 were assessed with a paired *t*-test or Wilcoxon’s signed rank test. The analysis of changes in the total and HMW APN and HMW/total APN in each group, adjusted for confounding factors, used a general linear model for repeated measurements. The changes in variables due to the administration of MSE 150 mg/day or 300 mg/day were compared with the placebo group using an analysis of covariance (ANCOVA). The changes in variables were measured as the unstandardized partial regression coefficient (Β) values with standard errors (SEs), which were adjusted for potentially confounding factors. Furthermore, the effects of the *DsbA-L* or *ADIPOQ* genotypes with the changes in the ratio of HMW/total APN due to MSE administration were evaluated by the ANCOVA after the stratification of participants by genotypes. Structural equation modeling was used to perform the pathway analysis assessing the effect of MSE administration on the APN multimerization in relation to the mRNA levels of APN multimerization-regulated genes (*i.e.* *DsbA-L, FoxO1*, *PPARγ*, *ERp-44*, *Ero1-Lα* and *AMPK*). The goodness of fit on the structural equation modeling was evaluated by the goodness of fit index (GFI), Bentler-Bonett normed fit index (NFI), comparative fit index (CFI) and root mean square error of approximation (RMSEA). In order to examine the accuracy of the parameters of the multivariable models and structural equation models, bootstrap analyses were performed using 1,000 replicated datasets generated by random sampling with replacement. A P value < 0.05 was statistically significant. The calculations of sample size, the structural modeling process and other statistical analyses were performed using the G*Power software program (version 3.1.9.2; Heinrich Heine University Düsseldorf, Düsseldorf, Germany), the SPSS Amos software program (version 23.0, IBM Japan Inc., Tokyo, Japan) and the SPSS software package (version 23.0, IBM Japan Inc.), respectively.

## Results

### Characteristics of the participants

In the human study, all of the participants completed the clinical trial according to the study protocol and took all of the test tablets that were dispensed. None of the participants experienced any adverse effects from the oral administration of test tablets during the clinical trial period.

The frequencies of the *DsbA-L* rs1917760 G/G, G/T and T/T genotypes were 50.0%, 45.2% and 4.8%, respectively; those of the *ADIPOQ* rs3774261 A/A, A/G and G/G genotypes were 35.7%, 50% and 14.3%, respectively; and those of the *ADIPOQ* rs182052 A/A, A/G and G/G genotypes were 26.2%, 52.4% and 21.4%, respectively. These observed genotype frequency distributions were consistent with the Hardy-Weinberg equilibrium (P > 0.05). The frequencies of these genotypes did not differ markedly among the MSE 150 mg/day, 300 mg/day and placebo groups (Supplementary Table S2). In the analyses of this study, since there were few participants with the *DsbA-L* T/T genotype (n = 2) or *ADIPOQ* rs3774261 G/G genotype (n = 6), the *DsbA-L* G/T and T/T genotypes and the *ADIPOQ* rs3774261 A/G and G/G genotypes were integrated. The demographic and clinical characteristics of the participants at the baseline are shown in Table 1. The demographic and clinical parameters did not differ among the three test tablet groups (Table 1).

**Table 1:** The demographic and clinical characteristics of the young, healthy volunteers at the baseline.

	Placebo (n = 14)	MSE 150 mg/day (n = 14)	MSE 300 mg/day (n = 14)	P
Age (years)	22.8 (20–26)	23.7 (21–46)	22.5 (20–25)	0.98^a^
Bodyweight (kg)	61.4 (50.0–80.0)	58.8 (44.0–70.0)	64.0 (52.0–87.0)	0.45^a^
BMI (kg/m^2^)	21.0 ± 3.4	20.7 ± 2.5	21.9 ± 2.6	0.52
AST (U/L)	17.9 ± 3.3	18.2 ± 2.4	18.9 ± 5.3	0.78
ALT (U/L)	16.8 ± 6.9	14.6 ± 4.8	19.2 ± 10.2	0.30
γ-GT (U/L)	21.3 ± 7.4	19.0 ± 6.9	21.0 ± 8.9	0.70
HDL-C (mg/dL)	62.1 ± 12.8	59.5 ± 9.1	58.3 ± 14.5	0.71
LDL-C (mg/dL)	99.0 ± 22.1	97.3 ± 22.3	109.3 ± 33.6	0.44
Triglyceride (mg/dL)	81.3 (35–235)	76.6 (40–166)	81.8 (37–201)	0.95^a^
Total protein (g/dL)	7.38 ± 0.31	7.23 ± 0.19	7.38 ± 0.34	0.29
Albumin (g/dL)	4.80 ± 0.20	4.81 ± 0.14	4.74 ± 0.24	0.53
Glycoalbumin (%)	13.3 (12.1-14.7)	13.2 (11.8-14.5)	13.0 (11.8-15.3)	0.54^a^
Oxidized albumin (%)	26.3 ± 3.3	27.7 ± 4.5	27.2 ± 7.9	0.81
Total bilirubin (mg/dL)	0.82 ± 0.31	0.94 ± 0.32	0.80 ± 0.43	0.53
Creatinine (mg/dL)	0.83 ± 0.065	0.82 ± 0.12	0.86 ± 0.10	0.54
BUN (mg/dL)	13.7 ± 2.8	12.8 ± 2.9	13.1 ± 3.8	0.74
Uric acid (mg/dL)	5.94 ± 1.01	5.79 ± 1.54	5.84 ± 1.11	0.95
Total APN (µg/mL)	6.00 (0.84–17.98)	6.42 (0.82–18.25)	8.26 (3.29–22.02)	0.61^a^
HMW APN (µg/mL)	2.95 (0.29–10.4)	3.17 (0.20–10.43)	4.31 (1.47–14.06)	0.68^a^
HMW/total APN (%)	49.1 (27.0–67.0)	49.4 (24.5–62.7)	52.2 (33.8–67.5)	0.90^a^
*DsbA-L* mRNA	0.93 (0.48–1.62)	0.84 (0.57–1.13)	0.91 (0.58–1.54)	0.37^a^
*AMPK* mRNA	0.66 (0.42–1.29)	0.67 (0.47–0.89)	0.66 (0.40–0.94)	0.79^a^
*FoxO1* mRNA	0.66 (0.31–1.18)	0.67 (0.46–0.98)	0.69 (0.36–1.25)	0.92^a^
*PPARγ* mRNA	1.26 (0.56–2.42)	1.12 (0.43–2.39)	1.45 (0.52–3.87)	0.52^a^
*ERp-44* mRNA	0.81 (0.51–1.23)	0.90 (0.62–1.33)	0.80 (0.63–1.03)	0.42^a^
*Ero1-Lα* mRNA	0.87 (0.54–1.61)	0.96 (0.67–1.44)	0.92 (0.63–1.17)	0.73^a^

The data are the means ± standard deviation or geometric mean (range).

^a^Kruskal-Wallis test (otherwise, one-way ANOVA was used).

MSE, melinjo seeds extract; BMI, body mass index; AST, aspartate aminotransferase; ALT, alanine aminotransferase; γ-GT, γ-glutamyl transferase; HDL-C, high-density lipoprotein cholesterol; LDL-C, low-density lipoprotein cholesterol; BUN, blood urea nitrogen; APN, adiponectin; DsbA-L, disulfide-bond A oxidoreductase-like protein; AMPK, 5′-Adenosine monophosphate-activated protein kinase; FoxO1, forkhead box protein O1; PPARγ, peroxisome proliferator-activated receptor γ; ERp-44, endoplasmic reticulum protein 44; Ero1-Lα, endoplasmic reticulum oxidoreductase 1-like protein *α*.

### The effect of MSE administration on APN multimerization in humans

First, we compared the levels of total APN, HMW APN and HMW/total APN at the endpoint between three test tablet groups (Supplementary Table S3). At the endpoint, the ratio of HMW/total APN in the MSE 300 mg/day group was higher than that in the other groups (Supplementary Table S3).

Second, we examined the changes in the levels of total APN, HMW APN and the ratio of HMW/total APN before and after the administration of test tablets in each group (Supplementary Table S3). The levels of total and HMW APN and the ratio of HMW/total APN tended to increase following the administration of the test tablets in MSE 150 mg/day and/or 300 mg/day groups, although the effects were not significant (Supplementary Table S3). In contrast, in the analyses with adjusting for confounding factors (*i.e.* BMI and glycoalbumin) using a general linear model for repeated measures, the total APN level was increased following the administration of the MSE 150 mg/day (Supplementary Table S4). The values of alanine aminotransferase (ALT) and low-density lipoprotein cholesterol (LDL-C) were significantly decreased by the administration of MSE 300 mg/day (Supplementary Table S3). No significant changes in other parameters, including oxidized HSA, were observed by the administration of the test tablets in each group (Supplementary Table S3).

**Figure 1:**
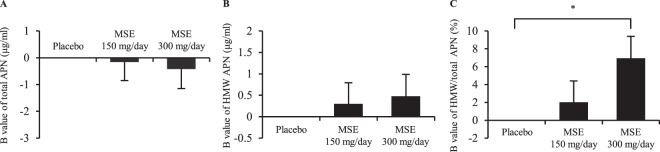
The association between the administration of MSE and the levels of total APN and HMW APN and the HMW/total APN ratio in healthy volunteers. The comparison of changes in the levels of total APN (**A**) and HMW APN (**B**) and the ratio of HMW/total APN (**C**) before and on day 14 after the administration of test tablets among the groups of placebo, MSE 150 mg/day and MSE 300 mg/day. P values were calculated by an ANCOVA (*P < 0.05). The bars and error bars represent the unstandardized partial regression coefficients (B values) and SEs, respectively. B values (SEs) and P values were adjusted by the BMI and glycoalbumin plus level of total APN (**A**) or HMW **A**PN (**B**) or ratio of HMW/total APN (**C**) at the baseline. MSE, melinjo seed extract; HMW, high-molecular-weight; APN, adiponectin; DsbA-L, disulfide bond A oxidoreductase-like protein; SE, standard error.

Third, we compared the changes in the levels of total APN, HMW APN and HMW/total APN before and after the administration of test tablets between the groups by an ANCOVA (Fig. 1 and Supplementary Table S5). The changes in the level of HMW APN and HMW/total APN correlated with their respective values at baseline (r = −0.380, P = 0.013; r = −0.673, P < 0.001, respectively). Therefore, each partial regression coefficient (B value) of the levels of total APN, HMW APN and ratio of HMW/total APN was adjusted by each value at baseline in addition to BMI and glycoalbumin (Fig. 1 and Supplementary Table S5). No significant differences in the levels of total APN or HMW APN following the administration of MSE 150 mg/day or 300 mg/day versus those following placebo administrations were observed (Fig. 1A,B and Supplementary Table S5). In contrast, in the analysis with adjusting by the BMI, glycoalbumin and ratio of HMW/total APN at baseline, the ratio of HMW/total APN was significantly increased by the administration of MSE 300 mg/day compared with the value following placebo administration (Fig. 1C and Supplementary Table S5). All 1000 bootstrap runs for the multivariable model regarding the ratio of HMW/total APN exhibited successful minimization and were included in the bootstrap analysis (Supplementary Table S6). The results of the bootstrap evaluation for this multivariable model showed that the bias values, SEs and 95% confidence intervals (CIs) for all covariates obtained using the bootstrap analysis were generally comparable to the estimates obtained using the ANCOVA (Supplementary Table S6). Supplementary Table S7 shows the changes in clinical parameters due to MSE administration according to the ANCOVA, and no significant effect of MSE administration on the clinical parameters was observed.

**Figure 2:**
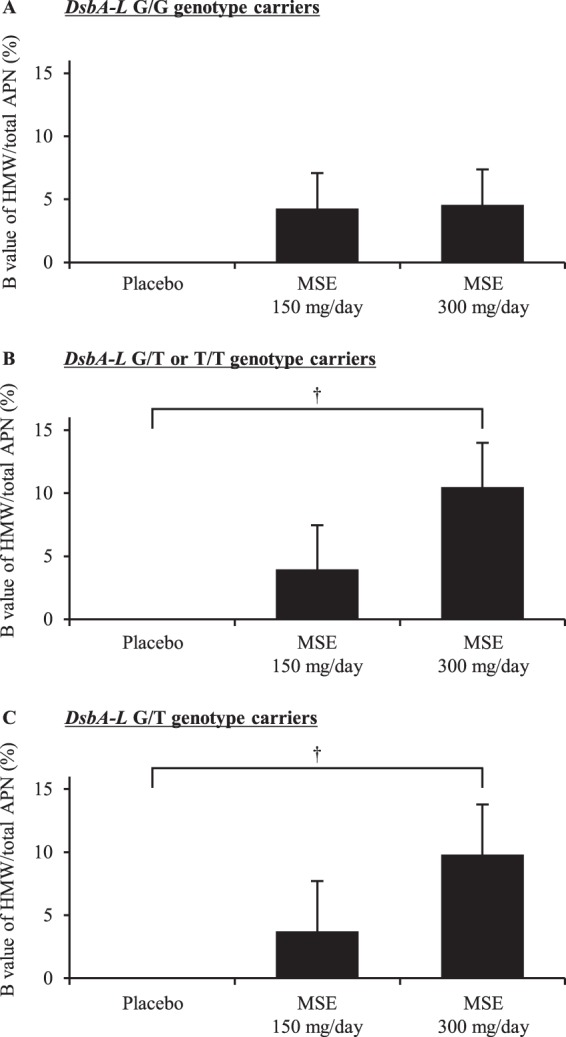
The association between the administration of MSE and the HMW/total APN ratio in healthy volunteers, with stratification by the *DsbA-L* genotype. The comparison of changes in the ratio of HMW/total APN before and on day 14 after the administration of test tablets among the groups of placebo, MSE 150 mg/day and MSE 300 mg/day in the carriers of the *DsbA-L* G/G genotype (**A**), in those of the *DsbA-L* G/T or T/T genotype (**B**) and in those of *DsbA-L* G/T genotype. (**C**) An association among carriers of the *DsbA-L* T/T genotype could not be detected due to the small sample size (n = 2). P values were calculated by an ANCOVA (^†^P < 0.01). The bars and error bars represent the unstandardized partial regression coefficients (B values) and SEs, respectively. B values (SEs) and P values were adjusted by the BMI, glycoalbumin and ratio of HMW/total APN at the baseline. MSE, melinjo seed extract; HMW, high-molecular-weight; APN, adiponectin; DsbA-L, disulfide bond A oxidoreductase-like protein; SE, standard error.

Fourth, we analyzed the effects of the *DsbA-L* or *ADIPOQ* genotypes on the change in the ratio of HMW/total APN due to MSE administration. The analyses stratified by the *DsbA-L* genotype showed that the changes in the ratio of HMW/total APN after the administration of MSE 300 mg/day were pronounced in carriers of the *DsbA-L* G/T or T/T genotypes and in carriers of the G/T genotype (Fig. 2 and Supplementary Table S6). All 1000 bootstrap runs exhibited successful minimization and were included in the bootstrap analysis. The bootstrap analyses for this multivariable models among the carriers of the *DsbA-L* G/T or T/T genotypes and those of G/T genotype showed that the bias values, SEs and 95% CIs for all covariates obtained using the bootstrap analyses were generally comparable to the estimates obtained using the ANCOVA (Supplementary Table S6). However, no significant difference in the ratio of HMW/total APN by the administration of MSE was observed in the carriers of the *DsbA-L* G/G genotype (Fig. 2 and Supplementary Table S6). The *ADIPOQ* genotypes seemed to have no marked influence on the change in the ratio of HMW/total APN due to MSE administration (Supplementary Fig. S3).

### Association between MSE administration and changes in the gene expression in humans

**Figure 3:**
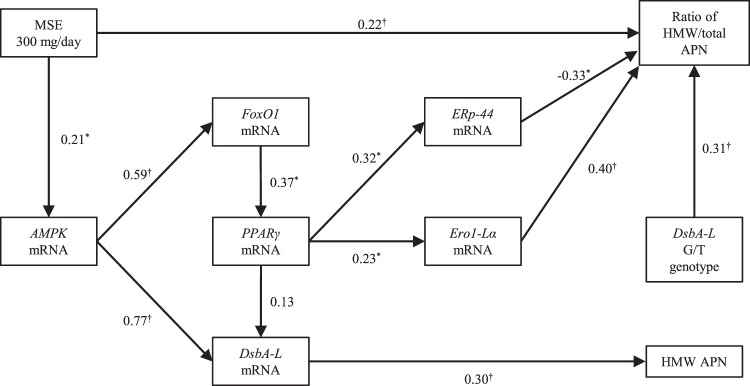
The structural equation modeling diagram of MSE administration to healthy volunteers and APN multimerization. Lines with numbers indicate significant paths with standardized partial regression coefficients (*P < 0.05, ^†^P < 0.01). Arrows represent an association between two factors. The β values ranged from −1 to 1, with a positive value representing a positive correlation and negative value representing a negative correlation. MSE, melinjo seed extract; APN, adiponectin; DsbA-L, disulfide-bond A oxidoreductase-like protein; AMPK, 5′-Adenosine monophosphate-activated protein kinase; FoxO1, forkhead box protein O1; PPARγ, peroxisome proliferator-activated receptor γ; ERp-44, endoplasmic reticulum protein 44; Ero1-Lα, endoplasmic reticulum oxidoreductase 1-like protein α; HMW high-molecular-weight.

To evaluate the relationships between MSE administration, APN multimerization and genes regulating the APN multimerization, we performed a pathway analysis using structural equation modeling (Fig. 3). The GFI, NFI, CFI and RMSEA of the constructed structural equation model were 0.913, 0891, 0.999 and < 0.001, respectively. Taken together, these fitness statistics indicated a good fit for the structural equation model. Furthermore, all 1000 bootstrap runs exhibited successful minimization and were included in the bootstrap analysis. The result of the bootstrap evaluation for this structural equation model showed that the bias values, SEs and 95% CIs for all covariates obtained using the bootstrap analysis were generally comparable to the estimates obtained using the structural equation modeling (Supplementary Table S8). The model showed that the administration of MSE 300 mg/day increased *DsbA-L* *via* *AMPK*, *FoxO1* and *PPARγ*, resulting in an increase in the level of HMW APN (Fig. 3). Furthermore, the model showed that the administration of MSE 300 mg/day increased *ERp-44* and *Ero1-Lα* *via* *AMPK*, *FoxO1* and *PPARγ*, resulting in a change in the ratio of HMW/total APN (Fig. 3), and also directly increased the ratio of HMW/total APN (Fig. 3). The model additionally showed that the *DsbA-L* rs1917760 polymorphism was associated with a change in the ratio of HMW/total APN by the administration of MSE 300 mg/day (Fig. 3). We further constructed a structural equation modeling diagram among carriers of the *DsbA-L* G/T or T/T genotypes and those of the G/T genotype (Supplementary Figs. S4 and S5). Of 1000 bootstrap runs among carriers of the *DsbA-L* G/T or T/T genotypes and carriers of the G/T genotype, 948 and 826 that exhibited successful minimization were included in the bootstrap analyses, respectively. The results of the bootstrap analyses and good fitness statistics indicated a slightly good fit for the structural equation models (Supplementary Tables S9 and S10). These models showed that the activating effect of the administration of MSE 300 mg/day on the genes regulating APN multimerization (*i.e.* *AMPK*, *FoxO1*, *PPARγ* and *DsbA-L*) might be more pronounced in the carriers of the *DsbA-L* G/T or T/T genotypes and those of the G/T genotype than in others (Supplementary Figs. S4 and S5). In contrast, the structural equation modeling diagram among the carriers of *DsbA-L* G/G genotype could not be constructed due to low goodness of fit.

### Effects of MSE administration on APN multimerization in mice

Given that the administration of MSE was shown to be associated with APN multimerization according to the results of the present randomized, double-blind placebo-controlled clinical trial conducted among Japanese healthy volunteers, we further examined the effects of MSE administration on the levels of APN under obese conditions in mice. We first examined the effects of MSE administration on control-diet-fed mice (Supplementary Figs. S6 and S7). MSE administration to control-diet-fed mice had no effect on the body weight or fat mass (Supplementary Fig. S6). Furthermore, MSE administration in control-diet-fed mice did not affect the expression of *DsbA-L* mRNA in adipose tissue (Supplementary Fig. S7A). In contrast, the administration of MSE 1,000 mg/kg/day to control-diet-fed mice increased the mRNA level of *ADIPOQ* in adipose tissue and the protein levels of total APN and HMW APN in serum (Supplementary Fig. S7B–D).

### Effects of MSE administration on obesity in mice

**Figure 4:**
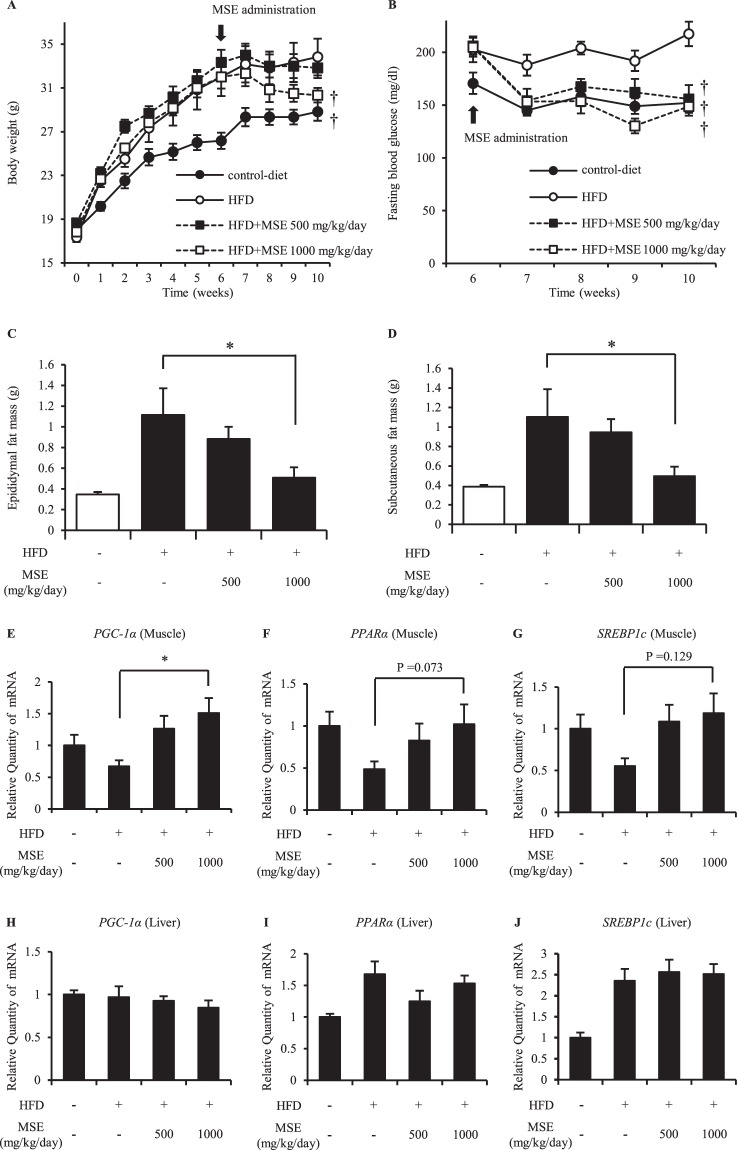
The effects of MSE administration to HFD mice on the weight status, fasting blood glucose and fat mass. (**A**) Body weight changes in HFD mice treated with vehicle or MSE (500 or 1,000 mg/kg/day). (**B**) Fasting blood glucose level in HFD mice treated with vehicle or MSE (500 or 1,000 mg/kg/day). (**C**,**D**) Epididymal fat mass level (**C**) and subcutaneous fat mass level (**D**) in HFD mice treated with vehicle or MSE (500 or1,000 mg/kg/day). (**E**–**J**) mRNA expression of *PGC-1α* (**E**, muscle; **H**, liver), *PPARγ* (**F**, muscle; **I**, liver) and *SREBP1c* (**G**, muscle; **J**, liver) in HFD mice treated with vehicle or MSE (500 or 1000 mg/kg/day). The blood glucose test was performed on 6- to 10-week-old HFD mice. Data are means ± standard errors; n = 6 mice/group. P values were calculated by Dunnett’s procedure (*P < 0.05, ^†^P < 0.01). MSE, melinjo seed extract; HFD, high-fat-diet; PGC-1α, peroxisome proliferator-activated receptor gamma coactivator 1α; PPARα, peroxisome proliferator-activated receptor α; SREBP1c, sterol regulatory element-binding protein 1c.

**Figure 5:**
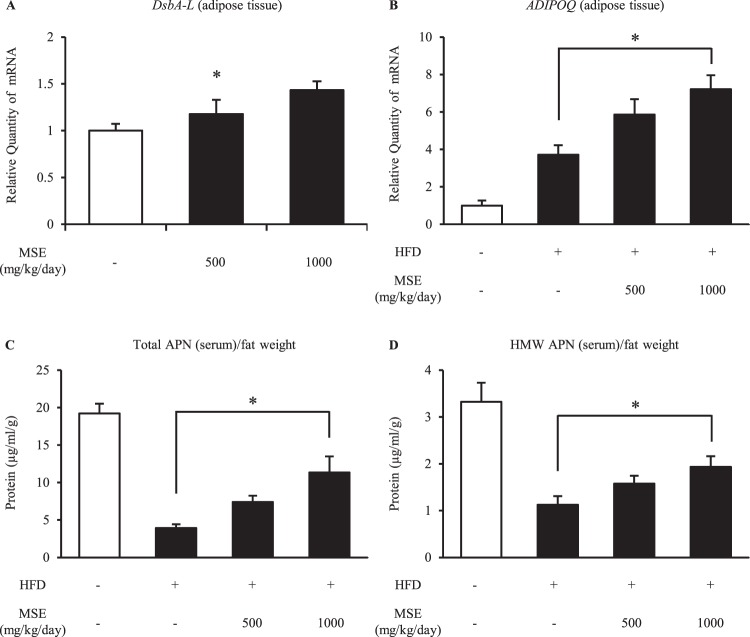
The effects of MSE administration to HFD mice on the mRNA expression of *DsbA-L*, *ADIPOQ* and protein levels of APN. (**A**) mRNA expression of *DsbA-L* (adipose tissue) in HFD mice treated with vehicle or MSE (500 or 1,000 mg/kg/day). (**B**) mRNA expression of *ADIPOQ* (adipose tissue) in HFD mice treated with vehicle or MSE (500 or 1,000 mg/kg/day). (**C**,**D**) Serum adiponectin levels adjusted to fat mass in control-diet-fed mice treated with vehicle or HFD mice treated with vehicle or MSE (500 or 1,000 mg/kg/day). Data are means ± standard errors; n = 6 mice/group. P values were calculated by Dunnett procedure (*P < 0.05, ^†^P < 0.01). MSE, melinjo seed extract; DsbA-L, disulfide-bond A oxidoreductase-like protein; ADIPOQ, adiponectin gene; APN, adiponectin; HMW, high-molecular-weight; HFD, high-fat-diet.

Next, we examined the effect of MSE administration in HFD mice (Figs. 4 and 5). In order to confirm obese conditions in the HFD mice, the weight status, blood glucose level and fat mass were measured (Fig. 4). The body weight, fasting blood glucose level, subcutaneous fat mass and epididymal fat mass increased predominantly due to HFD loading, and these increasing trends were suppressed by MSE administration (Fig. 4A–D). Moreover, we examined the effects of the administration of MSE on the metabolic performance markers in HFD mice (Fig. 4E–J). In muscle, the administration of MSE corrected the mRNA levels of dysregulated genes, such as *PGC-1α* as well as tended to improve the mRNA levels of *PPARα* and *SREBP1c* (Fig. 4E–G). However, these effects were not observed in the liver (Fig. 4H–J), indicating that MSE dominantly targets muscle to improve insulin resistance condition. An examination of the expression of *DsbA-L* mRNA in adipose tissue among HFD mice showed that the administration of MSE to HFD mice increased the expression of *DsbA-L* mRNA (Fig. 5A). In addition, the administration of MSE to HFD mice increased the mRNA level of *ADIPOQ* in adipose tissue and protein levels of total APN and HMW APN in serum (Fig. 5B–D).

## Discussion

This is the first study to show that the oral administration of MSE 300 mg/day was associated with an increase in the ratio of HMW/total APN among healthy Japanese volunteers, and this association was more pronounced than usual in carriers of the *DsbA-L* G/T or T/T genotypes. Furthermore, the structural equation model showed that the association between the administration of MSE 300 mg/day and the increase in the ratio of HMW/total APN was mediated by the *AMPK* signaling pathway including *DsbA-L*. In addition to the clinical trial under physiological conditions, in the mouse study, we confirmed that MSE increased the levels of total and HMW APN in the serum and suppressed the metabolic abnormalities (*i.e.* weight gain, increased blood glucose level and fat mass accumulation) in HFD mice. Furthermore, by administering MSE to HFD mice, we were able to determine the increased mRNA levels of *ADIPOQ* and *DsbA-L* in adipose tissue, a major tissue involved in APN multimerization. These findings suggest that MSE may enhance APN multimerization under physiological conditions (as observed in our clinical trial) and that the effects of MSE may also be conserved under obese conditions through APN multimerization in adipose tissue (as observed in our mouse *in vivo* studies).

APN has been shown to enhance insulin sensitivity and protect against obesity, type 2 diabetes and atherosclerosis^10–13^. HMW APN is considered the most active form of the protein^11,12^, and the ratio of HMW/total APN has been reported to be more closely correlated with central obesity and insulin resistance status than the total APN level. Conversely, LMW APN has been reported to be inversely correlated with the risk of type 2 diabetes^12^, so studies should focus on not only the total APN level but also the ratio of HMW/total APN. APN multimerization was reported to be regulated by post-transcription-dependent mechanisms involving ER chaperones, such as DsbA-L, Ero1-Lα or ERp44^16^. The overexpression of *DsbA-L* enhances APN multimerization in 3T3-L1 adipocytes^15^ and protects mice from HFD-induced hepatosteatosis and insulin resistance^29^. Furthermore, suppressing *DsbA-L* expression reduced APN levels and secretion in 3T3-L1 adipocytes^15^. The present randomized, double-blind placebo-controlled clinical trial showed that the administration of MSE to healthy volunteers was associated with an increase in the ratio of HMW/total APN in relation to *DsbA-L* (Figs. 1–3). Thus, even in relatively healthy individuals, the administration of MSE may have health benefits. On the other hand, our mouse study showed that the administration of MSE to HFD mice suppressed their increasing trends in body weight, fasting blood glucose and fat mass, and increased the APN levels and the mRNA expression of *ADIPOQ* and *DsbA-L* in adipose tissue (Figs. 4 and 5). Thus, the administration of MSE may be effective for preventing and/or treating obesity and its associated metabolic complications by promoting APN multimerization *via* DsbA-L, although further human studies in obese/overweight subjects are needed to verify these findings.

Our recent clinical study showed that the *DsbA-L* T/T genotype is involved in the increase in the body mass index (BMI) associated with the decrease in the rate of HMW/total APN among Japanese subjects who participated in a health screening program^26^. Our other study also showed that the *DsbA-L* T/T genotype is associated with an increased prevalence of overweight and decreased levels of *DsbA-L* mRNA in PBMCs among Japanese male schizophrenia patients^30^. Gao *et al*. previously showed that the *DsbA-L* rs1917760 polymorphism was associated with increased insulin secretion and fat deposition in a cross-sectional study conducted in a Chinese population^31^. Therefore, carriers of the *DsbA-L* rs1917760 polymorphism are considered to have a low expression of *DsbA-L* mRNA and to be at a high risk of developing obesity and insulin resistance. In the present study, the association between the administration of MSE and the ratio of HMW/total APN was more pronounced in *DsbA-L* rs1917760 T allele carriers than in non-carriers (Fig. 2 and Supplementary Table S6). Furthermore, the change in the ratio of HMW/total APN by MSE administration was inversely correlated with the ratio at the baseline. The effect of MSE administration on the APN multimerization may therefore be particularly pronounced among individuals with metabolic diseases or in high-risk groups thereof. Based on these findings, it may be better to administer MSE only to subjects in whom this treatment is likely to be most effective. Identifying the high-risk *DsbA-L* genotypes may be useful for preventing obesity, insulin resistance and related complications by facilitating targeted prevention and treatment programs including supplementation of MSE for individuals with high-risk genotypes. On the other hand, in the present study, the significant association between the administration of MSE and APN multimerization was only observed in *DsbA-L* G/T genotype carriers and T allele carriers (*i.e.* the G/T or T/T genotype carriers). Since the number of *DsbA-L* T/T genotype carriers was very limited (n = 2), we could not detect the association between MSE and APN multimerization in the T/T genotype carriers. A previous study reported that the *DsbA-L* T allele was associated with promotor activity, and the effect is thought to be more pronounced in the T/T genotype than in the G/T genotype^18^. Nevertheless, it is unclear whether the effect of MSE on APN multimerization was also more pronounced in the T/T genotype than in the G/T genotype.

Since DsbA-L was originally identified as a kappa class of glutathione S-transferase (GST) and is a renamed protein, it also has glutathione conjugation activity and is involved in the cellular detoxification of xenobiotics, endogenous toxic metabolites and free radicals^32^ Bai *et al*. recently showed that DsbA-L plays an important role in maintaining the mitochondrial integrity and protecting against inflammation and insulin resistance by activating the cGMP-AMP (cGAMP) synthase-cGAMP-stimulator of interferon genes pathway^33^. Therefore, activation of *DsbA-L* by MSE administration may exert beneficial effects not only *via* APN activation but also other molecular mechanisms.

The previous *in vitro* study demonstrated the up-regulation of DsbA-L, which is mediated by PPARγ, FoxO1 and AMPK signaling pathways^17^. APN multimerization is up-regulated by treatment with thiazolidinediones (TZDs), which stimulate DsbA-L activity *via* a PPARγ activation^16^. On the other hand, the stimulatory effect of resveratrols on DsbA-L activity was reported to be regulated by the FoxO1 and the AMPK signaling pathways including PPARγ activation^16^. In the present study, the structural equation model showed for the first time in humans that the oral dose of MSE 300 mg/day increased the HMW APN level and the ratio of HMW/total APN *via* *FoxO1* and *AMPK* signaling pathways (Fig. 3). Furthermore, *DsbA-L* was increased through *AMPK* and *PPARγ* (*via* *FoxO1*) and was involved in the increase in the HMW APN level (Fig. 3). However, the sample size and study period might have been insufficient to detect changes in the gene expressions induced by the administration of MSE because the sample size and study period of the clinical trial were determined based on detecting the effect of MSE on APN multimerization. Further investigations over longer periods in larger populations are needed to validate the present findings.

In the adjusted analyses using a general linear model for repeated measures, the total APN level was increased in the group receiving MSE 150 mg/day (Supplementary Table S4). However, in the adjusted analysis using an analysis of covariance, MSE 150 mg/day did not significantly increase the total APN level compared to placebo (Supplementary Table S5). On the other hand, the mouse study showed that the administration of MSE to HFD mice increased both the total APN and HMW APN levels (Fig. 5), suggesting that the administration of MSE might have activated APN synthesis as well as APN multimerization in the mouse study. Indeed, DsbA-L has been reported to be associated with not only APN multimerization but also the synthesis of APN^15^. Aside from species differences (the rate of HMW/total APN is higher in mice than in humans^11^), differences in the dosage, administration period and disease condition might have caused the observed difference in results between our human and mouse studies.

In the present study with control-diet-fed mice, MSE administration did not affect the expression of *DsbA-L* mRNA but increased the levels of total APN and HMW APN (Supplementary Fig. S7). Therefore, MSE partially affects the activation of APN though mechanisms other than *DsbA-L*. Indeed, a recent experimental study showed that MSE induced uncoupling protein 1-mediated thermogenesis in brown adipose tissue *via* the induction of sirtuin 1, resulting in the suppressions of adipose tissue inflammation and insulin resistance^34^. Furthermore, previous studies have shown that resveratrols have a range of effects, including the activation of Smad signaling, Fas and adenyl-cyclase pathways and the inhibition of transforming growth factor β1, nuclear factor kappa B, activator protein-1 and mitogen-activated protein kinase pathways^35,36^.

The main components of MSE used in the present study are dimers of trans-resveratrol (*i.e.* gnetin C, L, gnemonoside A, C and D). Since the bioavailability of trans-resveratrol is low and its retention time in blood shorter than that of MSE^1,5^, we speculated that the effect of MSE on APN multimerization mainly involved the dimers of trans-resveratrol contained in MSE, particularly gnetin C, which is rich in MSE. However, MSE contains trans-resveratrol itself, albeit in a small amount (about 0.1% in MSE). In order to clarify the active ingredients of MSE, it will be necessary to investigate whether or not the dimers of trans-resveratrol themselves are involved in APN multimerization.

The present human study showed that the values of ALT and LDL-C were decreased after the administration of MSE 300 mg/day, although these changes were within the normal range (Supplementary Table S3). The liver is the major target organ of APN and plays an important role in the insulin-sensitizing effect of APN^10,11^ APN is associated with decreased hepatic lipogenesis and increased β-oxidation^10^. Therefore, the enhancement effect of APN multimerization by MSE administration might be associated with a decrease in the values of ALT and LDL-C. However, the reductions in ALT and LDL-C due to MSE administration were not more significant than those noted with placebo administration (Supplementary Table S7). Of note, Konno *et al*. reported that the oral administration of 750 mg/day MSE for 8 weeks reduced the serum uric acid level and increased the high-density lipoprotein cholesterol level in healthy male volunteers in their double-blind, randomized placebo-controlled study^6^. Therefore, in the present human study, the effects of MSE administration on clinical parameters may not have been detected because of our subjects being young and healthy (23.2 ± 4.0 years), the short administration period (14 days) and the relatively low dosage involved (MSE 150 mg/day or 300 mg/day). Further investigations over longer periods, with a wider range of doses and more diverse background populations (*e.g.* middle-aged subjects and subjects with obesity, fatty liver disease, dyslipidemia and gout) are therefore needed.

## Conclusions

**Figure 6:**
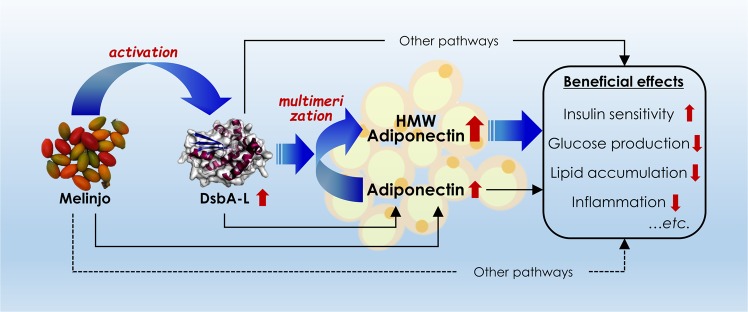
Proposed mechanisms by which melinjo activates adiponectin. DsbA-L, disulfide-bond A oxidoreductase-like protein; HMW, high-molecular-weight.

The present randomized, double-blind placebo-controlled clinical study showed that the administration of MSE was associated with an increase in the ratio of HMW/total APN, with an even greater association than usual noted in subjects with the *DsbA-L* rs1917760 T allele. Furthermore, we showed that the administration of MSE to HFD mice suppressed the weight gain and the increases in the fasting blood glucose levels and fat mass accumulation in relation to APN multimerization. When we combined the results from human and mouse studies, the findings suggested that MSE might be an effective functional food for individuals at a high risk of developing obesity and related metabolic diseases (Fig. 6), although further studies will be needed to verify the present findings and investigate the beneficial effects of MSE on the development and progression of obesity and its related metabolic diseases in humans.

## Supplementary information

Supplementary Information.

## Data Availability

The datasets generated during and/or analyzed during the current study are available from the corresponding author on reasonable request.
