# Supplementary material for: Melinjo seed extract increases adiponectin multimerization in physiological and pathological conditions
Source: Sci Rep. 2020 Mar 9;10:4313. doi: 10.1038/s41598-020-61148-2 (PMC7062855; doi:10.1038/s41598-020-61148-2)
Supplement: Supplementary file 1 — Supplementary Information. [file 41598_2020_61148_MOESM1_ESM.pdf]

Supplementary Information

Melinjo seed extract increases adiponectin multimerization in physiological and pathological conditions

Kentaro Oniki, Taisei Kawakami, Azusa Nakashima, Keishi Miyata, Takehisa Watanabe, Haruka Fujikawa, Ryunosuke Nakashima, Aoi Nasu, Yuka Eto, Noriki Takahashi, Hirofumi Nohara, Mary Ann Suico, Shunsuke Kotani, Yui Obata, Yuki Sakamoto, Yuri Seguchi, Junji Saruwatari, Tadashi Imafuku, Hiroshi Watanabe, Toru Maruyama, Hirofumi Kai, Tsuyoshi Shuto

Supplementary Table S1. Sequences of primers for quantitative RT-PCR.

Primer	Sequence
DsbA-L-forward	5'-GGTCCTATGCAGATACCAACAC-3'
DsbA-L-reverse	5'-GTACTGGCCTTTTCGGGGAA-3'
ADIPOQ-forward	5'-AAGGACAAGGCCGTTCTCT-3'
ADIPOQ-reverse	5'-GTTGACTGACGTTGATGGGTAT-3'
PGC-1α-forward	5'-TATGGAGTGACATAGAGTGTGCT-3'
PGC-1α-forward	5'-CCACTTCAATCCACCCAGAAAG-3'
PPARα-forward	5'-TATTCGGCTGAAGCTGGTGTAC-3'
PPARα-reverse	5'-CTGGCATTTGTTCCGGTTCT-3'
SREBP1c-forward	5'-CCCTTGACTTCCTTGCTGCA -3'
SREBP1c-reverse	5'-GCGTGAGTGTGGGCGAATC-3'
GAPDH-forward	5'-CCTGGAGAAACCTGCCAAGTATG-3'
GAPDH-reverse	5'-GGTCCTCAGTGTAGCCCAAGATG-3'

RT, reverse transcription; DsbA-L, disulfide bond A oxidoreductase-like protein; ADIPOQ, adiponectin gene; PGC-1α, peroxisome proliferator-activated receptor gamma coactivator 1α; PPARα, peroxisome proliferator-activated receptor α; SREBP1c, sterol regulatory element-binding protein 1c.

Supplementary Table S2. Frequencies of the *DsbA-L* and *ADIPOQ* genotypes in the young, healthy volunteers.

Genotypes		Placebo (n =14)	MSE 150 mg/day (n =14)	MSE 300 mg/day (n =14)	P
DsbA-L rs1917760	G/G	7 (50.0)	7 (50.0)	7 (50.0)	0.56
	G/T	6 (42.9)	7 (50.0)	6 (42.9)	
	T/T	1 (7.1)	0 (0.0)	1 (7.1)	
ADIPOQ rs3774261	A/A	4 (28.6)	6 (42.9)	5 (35.7)	0.45
	A/G	10 (71.4)	4 (28.6)	7 (50.0)	
	G/G	0 (0.0)	4 (28.6)	2 (14.3)	
ADIPOQ rs182052	A/A	3 (21.4)	5 (35.7)	3 (21.4)	0.25
	A/G	6 (42.9)	7 (50.0)	9 (64.3)	
	G/G	5 (35.7)	2 (14.3)	2 (14.3)	

The data are the number (%). P values were calculated by chi-square test. DsbA-L, disulfide bond A oxidoreductase-like protein; ADIPOQ, adiponectin gene; MSE, melinjo seeds extract.

Supplementary Table S3. Changes in parameters by the administration of MSE 150 mg/day, MSE 300 mg/day or placebo in the young, healthy volunteers.

	Placebo (n = 14)			MSE 150 mg/day (n =14)			MSE 300 mg/day (n =14)			P[a]
	Baseline	Day 14	P	Baseline	Day 14	P	Baseline	Day 14	P	
AST (U/L)	17.9 ± 3.3	18.1 ± 4.6	0.79[b]	18.2 ± 2.4	18.6 ± 2.5	0.52[b]	18.9 ± 5.3	17.8 ± 4.42	0.20	0.87[d]
ALT (U/L)	16.8 ± 6.9	16.4 ± 7.4	0.69[b]	14.6 ± 4.8	14.2 ± 4.4	0.56 [b]	19.2 ± 10.2	16.9 ± 9.2	0.01	0.59[d]
γ-GT (U/L)	21.3 ± 7.4	21.1 ± 8.2	0.71[b]	19.0 ± 6.9	18.6 ± 5.9	0.47 [b]	21.0 ± 8.9	20.4 ± 7.4	0.40	0.65[d]
HDL-C (mg/dL)	62.1 ± 12.8	61.3 ± 14.2	0.57[b]	59.5 ± 9.1	58.9 ± 12.2	0.68 [b]	58.3 ± 14.5	55.6 ± 12.7	0.22	0.52[d]
LDL-C (mg/dL)	99.0 ± 22.1	95.1 ± 26.4	0.18[b]	97.3 ± 22.3	93.8 ± 25.1	0.22 [b]	109.3 ± 33.6	101.4 ± 29.5	0.05	0.73[d]
Triglyceride (mg/dL)	81.3 (35–235)	95.7 (36–220)	0.13[c]	76.6 (40–166)	106.2 (48–306)	0.08[c]	81.8 (37–201)	107.6 (51–456)	0.17[a]	0.97[e]
Total protein (g/dL)	7.38 ± 0.31	7.50 ± 0.20	0.13[b]	7.23 ± 0.19	7.21 ± 0.38	0.87 [b]	7.38 ± 0.34	7.34 ± 0.27	0.71	0.05[d]
Albumin (g/dL)	4.80 ± 0.20	4.85 ± 0.16	0.21[b]	4.81 ± 0.14	4.84 ± 0.27	0.68 [b]	4.74 ± 0.24	4.73 ± 0.25	0.92	0.32[d]
Glycoalbumin (%)	13.3 (12.1-14.7)	13.5 (12.1-14.6)	0.04[c]	13.2 (11.8-14.5)	13.4 (12.0-14.8)	0.13[c]	13.0 (11.8-15.3)	13.3 (11.9-14.9)	0.06[a]	0.71[e]
Oxidized albumin (%)	26.3 ± 3.3	27.4 ± 2.1	0.21[b]	27.7 ± 4.5	28.0 ± 3.1	0.68 [b]	27.2 ± 7.9	28.0 ± 7.8	0.54	0.94[d]
Total bilirubin (mg/dL)	0.82 ± 0.31	0.76 ± 0.16	0.39[b]	0.94 ± 0.32	0.98 ± 0.47	0.73 [b]	0.80 ± 0.43	0.76 ± 0.33	0.59	0.18[d]
Creatinine (mg/dL)	0.83 ± 0.065	0.83 ± 0.08	0.72[b]	0.82 ± 0.12	0.81 ± 0.11	0.69 [b]	0.86 ± 0.10	0.85 ± 0.11	0.29	0.70[d]
BUN (mg/dL)	13.7 ± 2.8	14.0 ± 2.1	0.59[b]	12.8 ± 2.9	12.2 ± 3.7	0.58 [b]	13.1 ± 3.8	13.2 ± 2.43	0.91	0.25[d]
Uric acid (mg/dL)	5.94 ± 1.01	6.24 ± 1.03	0.06[b]	5.79 ± 1.54	5.73 ± 1.29	0.77 [b]	5.84 ± 1.11	5.9 ± 1.25	0.44	0.54[d]
Total APN (μg/mL)	6.00 (0.84–17.98)	6.51 (1.46–21.05)	0.15[c]	6.42 (0.82–18.25)	7.04 (1.27–20.23)	0.06[c]	8.26 (3.29–22.02)	8.48 (3.39–18.82)	1.00[a]	0.61[e]
HMW APN (μg/mL)	2.95 (0.29–10.4)	3.33 (0.66–11.70)	0.63[c]	3.17 (0.20–10.43)	3.70 (0.40–11.84)	0.12[c]	4.31 (1.47–14.06)	4.89 (1.52–9.59)	0.12[a]	0.37[e]
HMW/total APN (%)	49.1 (27.0–67.0)	51.1 (43.9–59.1)	0.86[c]	49.4 (24.5–62.7)	52.5 (31.4–69.6)	0.14[c]	52.2 (33.8–67.5)	57.7 (40.9–70.6)	0.09[a]	0.02[e]

The data are the means ± standard deviation or geometric mean (range). [a] Comparison of parameters at the endpoint among the 3 groups.
[b] Paired *t*-test. [c] Wilcoxon signed rank test. [d] One-way ANOVA. [e] Kruskal-Wallis test. MSE, melinjo seeds extract; BMI, body mass index; AST, aspartate aminotransferase; ALT, alanine aminotransferase; γ-GT, γ-glutamyl transferase; BUN, blood urea nitrogen; HDL-C, high-density lipoprotein cholesterol; LDL-C, low-density lipoprotein cholesterol; APN, adiponectin; HMW, high molecular weight.

Supplementary Table S4. Changes in total and HMW APN and HMW / total APN for each group in young and healthy volunteers, determined using a general linear model of repeated measures.

	Mean ± SD		Adjusted analyses 1[a]	Adjusted analyses 2[b]
	Baseline	Day 14	P	P
Placebo (n = 14)				
Total APN (µg/mL)	7.55 ± 4.80	8.25 ± 5.62	0.082	0.087
HMW APN (µg/mL)	4.19 ± 3.10	4.38 ± 3.22	0.377	0.396
HMW/total APN (%)	50.6 ± 11.6	51.3 ± 4.3	0.700	0.706
MSE 150 mg/day (n =14)				
Total APN (µg/mL)	8.06 ± 5.07	8.71 ± 5.53	0.018	0.020
HMW APN (µg/mL)	4.37 ± 3.08	4.89 ± 3.38	0.089	0.062
HMW/total APN (%)	50.5 ± 9.3	53.2 ± 8.4	0.194	0.108
MSE 300 mg/day (n =14)				
Total APN (µg/mL)	9.51 ± 5.28	9.53 ± 4.68	0.978	0.971
HMW APN (µg/mL)	5.28 ± 3.64	5.51 ± 2.45	0.708	0.679
HMW/total APN (%)	53.0 ± 9.1	58.3 ± 8.3	0.110	0.127

[a] Adjusted by BMI at the baseline. [b] Adjusted by BMI and glycoalbumin at the baseline. HMW, high molecular weight; APN, adiponectin; MSE, melinjo seeds extract; SD, standard deviation; BMI, body mass index.

Supplementary Table S5. The association between the administration of MSE 150 mg or 300 mg and the total and HMW APN and ratio of HMW/total APN in young, healthy volunteers, as determined using an ANCOVA.

	Adjusted Model 1[a]			Adjusted Model 2[b]			Adjusted Model 3		
	B	SE	P	B	SE	P	B	SE	P
Total APN									
Placebo	0	-	-	0	-	-	0	-	-
MSE 150 mg/day	-0.09	0.76	0.903	-0.19	0.69	0.786	-0.16	0.69	0.819[c]
MSE 300 mg/day	-0.49	0.76	0.522	-0.63	0.70	0.373	-0.42	0.73	0.569[c]
HMW APN									
Placebo	0	-	-	0	-	-	0	-	-
MSE 150 mg/day	0.31	0.56	0.581	0.28	0.55	0.620	0.30	0.49	0.538[d]
MSE 300 mg/day	0.10	0.56	0.862	0.05	0.56	0.929	0.48	0.51	0.355[d]
HMW/total APN									
Placebo	0	-	-	0	-	-	0	-	-
MSE 150 mg/day	2.27	3.34	0.500	2.50	3.30	0.453	2.01	2.40	0.407[e]
MSE 300 mg/day	3.79	3.37	0.267	4.11	3.32	0.224	6.94	2.47	0.008[e]

[a] Adjusted by the BMI at the baseline. [b] Adjusted by the BMI and glycoalbumin at the baseline. [c] Adjusted by the BMI, glycoalbumin and total APN at the baseline. [d] Adjusted by the BMI, glycoalbumin and HMW APN at the baseline. [e] Adjusted by the BMI, glycoalbumin and HMW/total APN at the baseline. MSE, melinjo seeds extract; HMW, high molecular weight; APN, adiponectin; ANCOVA, analysis of covariance; B, unstandardized partial regression coefficient; SE, standard error; CI, confidence interval; BMI, body mass index.

Supplementary Table S6. The association between the administration of MSE and the ratio of HMW/total APN in young, healthy volunteers, as determined using an ANCOVA and a bootstrap analysis.

	ANCOVA			Bootstrap evaluation			
	B	SE	P	Bias	SE	95% CI	P
All participants (n =42)							
Placebo	0	-	-	-	-	-	-
MSE 150 mg/day	2.01	2.40	0.407	0.141	1.92	-1.75, 5.97	0.320
MSE 300 mg/day	6.94	2.47	0.008	0.261	2.59	1.86, 12.36	0.019
DsbA-L G/G carriers (n =21)							
Placebo	0	-	-	-	-	-	-
MSE 150 mg/day	4.26	2.83	0.153	-0.189	2.61	-0.86, 9.36	0.126
MSE 300 mg/day	4.55	2.81	0.126	0.474	3.08	-0.81, 11.29	0.152
DsbA-L G/T or T/T carriers (n =21)							
Placebo	0	-	-	-	-	-	-
MSE 150 mg/day	3.94	3.53	0.283	0.251	3.43	-1.62, 11.59	0.230
MSE 300 mg/day	10.49	3.50	0.009	-0.078	3.70	2.60, 17.73	0.015
DsbA-L G/T genotype carriers (n =19)							
Placebo	0	-	-	-	-	-	-
MSE 150 mg/day	3.74	3.98	0.364	-0.344	6.23	-8.17, 15.96	0.350
MSE 300 mg/day	9.80	3.99	0.029	0.231	5.68	-1.06, 21.74	0.063

Adjusted by the BMI, glycoalbumin and HMW/total APN at the baseline. An association among carriers of the *DsbA-L* T/T genotype could not be detected due to the small sample size (n = 2). MSE, melinjo seeds extract; HMW, high molecular weight; APN, adiponectin; ANCOVA, analysis of covariance; B, unstandardized partial regression coefficient; SE, standard error; CI, confidence interval; BMI, body mass index.

Supplementary Table S7. The changes in clinical parameters induced by MSE administration compared with those induced by placebo administration in the young, healthy volunteers using an ANCOVA.

	Placebo (n =14)	MSE 150 mg/day (n =14)			MSE 300 mg/day (n =14)		
	B	B	SE	P	B	SE	P
AST (U/L)	0	0.14	1.04	0.89	-1.36	1.04	0.20
ALT (U/L)	0	0.00	1.18	1.00	-1.86	1.18	0.12
γ-GT (U/L)	0	-0.21	0.79	0.79	-0.50	0.79	0.53
HDL-C (mg/dL)	0	0.21	2.29	0.93	-1.86	2.29	0.42
LDL-C (mg/dL)	0	0.43	4.35	0.92	-4.00	4.35	0.36
Triglyceride (mg/dL)	0	23.1	26.5	0.39	28.0	26.5	0.30
Total protein (g/dL)	0	-0.14	0.12	0.27	-0.16	0.12	0.20
Albumin (g/dL)	0	-0.029	0.075	0.71	-0.057	0.075	0.45
Glycoalbumin (%)	0	0.007	0.140	0.96	0.029	0.140	0.84
Oxidized albumin (%)	0	-0.75	1.37	0.59	-0.36	1.37	0.80
Total bilirubin (mg/dL)	0	0.093	0.11	0.41	0.021	0.11	0.85
Creatinine (mg/dL)	0	-0.013	0.022	0.57	-0.020	0.022	0.37
BUN (mg/dL)	0	-0.86	1.08	0.43	-0.23	1.08	0.83
Uric acid (mg/dL)	0	-0.36	0.25	0.16	-0.16	0.25	0.52

MSE, melinjo seeds extract; ANCOVA, analysis of covariance; B, unstandardized partial regression coefficient; SE, standard error; BMI, body mass index; AST, aspartate aminotransferase; ALT, alanine aminotransferase; γ-GT, γ-glutamyl transferase; BUN, blood urea nitrogen; HDL-C, high-density lipoprotein cholesterol; LDL-C, low-density lipoprotein cholesterol.

Supplementary Table S8. The bootstrap analysis of the structural equation model among the young, healthy volunteers.

	Structural equation modeling			Bootstrap evaluation			
	B	SE	P	Bias	SE	95% CI	P
AMPK mRNA ← MSE 300 mg/day	0.098	0.040	0.016	-0.001	0.057	0.002, 0.232	0.043
HMW/total APN ← MSE 300 mg/day	0.043	0.020	0.030	0.000	0.026	-0.010, 0.096	0.094
FoxO1 mRNA ← *AMPK* mRNA	0.474	0.098	<0.001	0.012	0.120	0.264, 0.747	0.003
DsbA-L mRNA ← *AMPK* mRNA	0.962	0.118	<0.001	0.012	0.100	0.749, 1.160	0.004
PPARγ mRNA ← *FoxO1* mRNA	4.406	1.718	0.010	0.083	2.044	0.292, 8.457	0.034
DsbA-L mRNA ← *PPARγ* mRNA	0.016	0.012	0.185	0.001	0.015	-0.008, 0.050	0.191
ERp-44 mRNA← *PPARγ* mRNA	0.041	0.018	0.020	0.002	0.018	0.012, 0.081	0.007
ERO1-Lα mRNA ← *PPARγ* mRNA	0.026	0.010	0.010	0.001	0.010	0.002, 0.045	0.029
HMW APN ← *DsbA-L* mRNA	1.556	0.629	0.013	-0.015	0.836	0.180, 3.445	0.025
HMW/total APN ← *ERp-44* mRNA	-0.108	0.044	0.014	0.000	0.048	-0.224, -0.036	0.007
HMW/total APN ← *ERO1-Lα* mRNA	0.144	0.049	0.003	0.003	0.048	0.062, 0.244	0.006
HMW/total APN← *DsbA-L* G/T genotype	0.056	0.019	0.004	0.000	0.021	0.017, 0.100	0.002

B, partial regression coefficient; SE, standard error; CI, confidence interval AMPK, 5'-Adenosine monophosphate-activated protein kinase; MSE, melinjo seeds extract; HMW high molecular weight; APN, adiponectin; FoxO1, forkhead box protein O1; DsbA-L, disulfide-bond A oxidoreductase-like protein; PPARγ, peroxisome proliferator-activated receptor γ; ERp-44, endoplasmic reticulum protein 44; Ero1-Lα, endoplasmic reticulum oxidoreductase 1-like protein α.

Supplementary Table S9. The bootstrap analysis of the structural equation model among the young, healthy volunteers with the *DsbA-L* G/T or T/T genotype.

	Structural equation modeling			Bootstrap evaluation			
	B	SE	P	Bias	SE	95% CI	P
AMPK mRNA ← MSE 300 mg/day	0.201	0.064	0.002	0.008	0.109	-0.019, 0.398	0.065
HMW/total APN ← MSE 300 mg/day	0.069	0.023	0.002	0.002	0.045	-0.019, 0.162	0.069
FoxO1 mRNA ← *AMPK* mRNA	0.364	0.096	<0.001	0.017	0.096	0.225, 0.638	0.002
DsbA-L mRNA ← *AMPK* mRNA	0.977	0.151	<0.001	0.031	0.159	0.728, 1.386	0.002
PPARγ mRNA ← *FoxO1* mRNA	6.027	2.517	0.017	-0.334	2.876	-0.100, 11.351	0.057
ERp-44 mRNA← *PPARγ* mRNA	0.049	0.023	0.034	0.002	0.030	0.002, 0.116	0.037
ERO1-Lα mRNA ← *PPARγ* mRNA	0.032	0.017	0.060	0.004	0.028	-0.014, 0.096	0.176
HMW APN ← *DsbA-L* mRNA	1.923	0.661	0.004	-0.155	1.139	-0.804, 3.548	0.138
HMW/total APN ← *ERp-44* mRNA	-0.113	0.058	0.054	0.011	0.104	-0.332, 0.087	0.264
HMW/total APN ← *ERO1-Lα* mRNA	0.211	0.050	<0.001	-0.014	0.095	0.031, 0.391	0.031

DsbA-L, disulfide-bond A oxidoreductase-like protein; B, partial regression coefficient; SE, standard error; CI, confidence interval AMPK, 5'-Adenosine monophosphate-activated protein kinase; MSE, melinjo seeds extract; HMW high molecular weight; APN, adiponectin; FoxO1, forkhead box protein O1; PPARγ, peroxisome proliferator-activated receptor γ; ERp-44, endoplasmic reticulum protein 44; Ero1-Lα, endoplasmic reticulum oxidoreductase 1-like protein α.

Supplementary Table S10. The bootstrap analysis of the structural equation model among the young, healthy volunteers with the *DsbA-L* G/T genotype.

	Structural equation modeling			Bootstrap evaluation			
	B	SE	P	Bias	SE	95% CI	P
AMPK mRNA ← MSE 300 mg/day	0.207	0.059	0.002	0.004	0.125	-0.077, 0.428	0.125
HMW/total APN ← MSE 300 mg/day	0.066	0.022	0.003	-0.005	0.050	-0.052, 0.148	0.183
FoxO1 mRNA ← *AMPK* mRNA	0.320	0.096	<0.001	0.010	0.085	0.163, 0.505	0.002
DsbA-L mRNA ← *AMPK* mRNA	0.978	0.152	<0.001	0.022	0.150	0.742, 1.335	0.002
PPARγ mRNA ← *FoxO1* mRNA	4.716	2.753	0.087	0.111	3.407	-0.469, 12.166	0.095
ERp-44 mRNA← *PPARγ* mRNA	0.068	0.026	0.008	0.004	0.035	0.014, 0.145	0.022
ERO1-Lα mRNA ← *PPARγ* mRNA	0.054	0.016	<0.001	0.005	0.029	0.008, 0.117	0.034
HMW APN ← *DsbA-L* mRNA	2.036	0.703	0.004	-0.136	1.075	-0.241, 3.657	0.084
HMW/total APN ← *ERp-44* mRNA	-0.118	0.062	0.055	0.024	0.112	-0.316, 0.108	0.333
HMW/total APN ← *ERO1-Lα* mRNA	0.219	0.051	<0.001	-0.018	0.094	0.040, 0.398	0.027

DsbA-L, disulfide-bond A oxidoreductase-like protein; B, partial regression coefficient; SE, standard error; CI, confidence interval AMPK, 5'-Adenosine monophosphate-activated protein kinase; MSE, melinjo seeds extract; HMW high molecular weight; APN, adiponectin; FoxO1, forkhead box protein O1; PPARγ, peroxisome proliferator-activated receptor γ; ERp-44, endoplasmic reticulum protein 44; Ero1-Lα, endoplasmic reticulum oxidoreductase 1-like protein α.

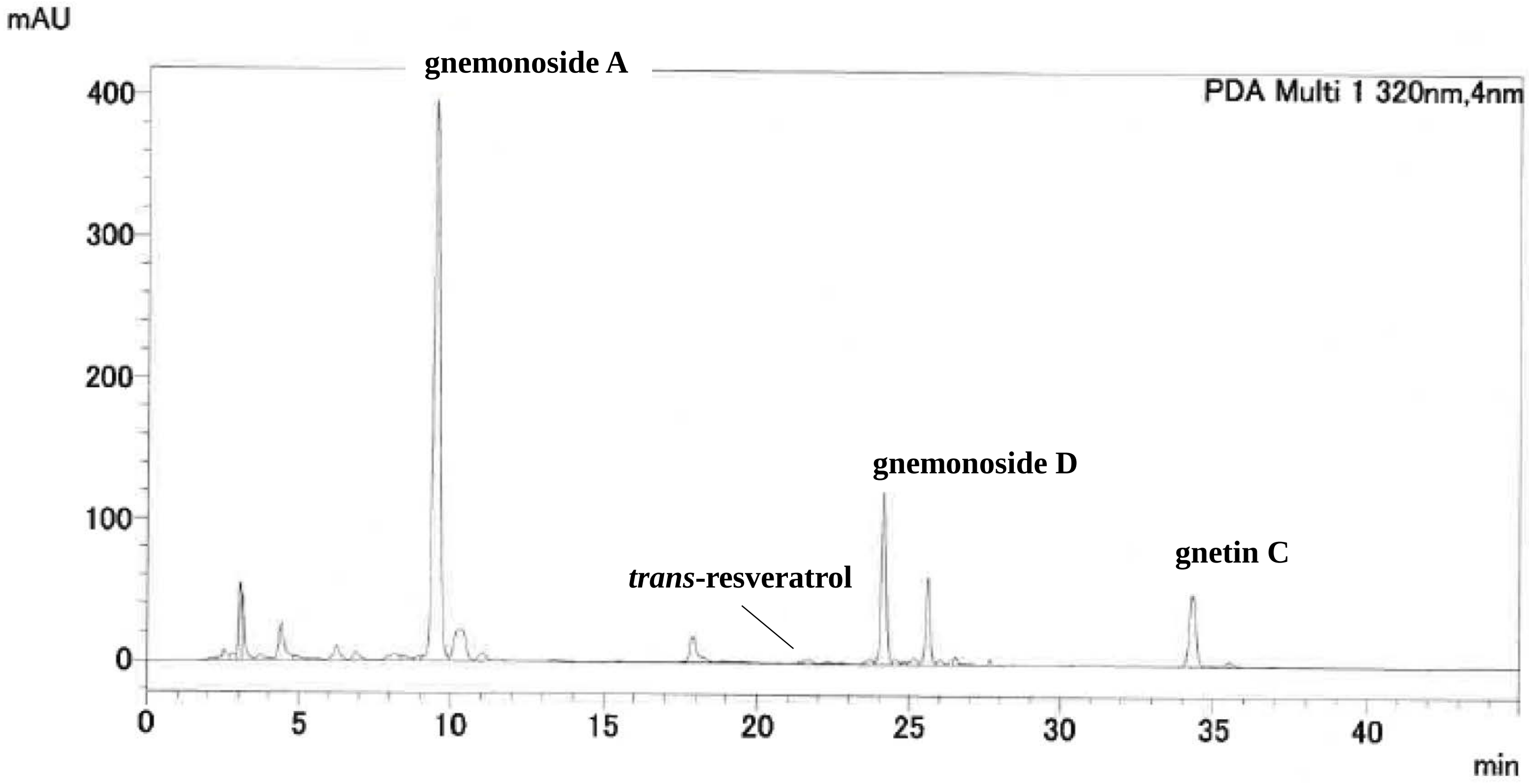

Supplementary Figure S1. The contents of resveratrol derivatives in MSE powder analyzed by high-performance liquid chromatography.

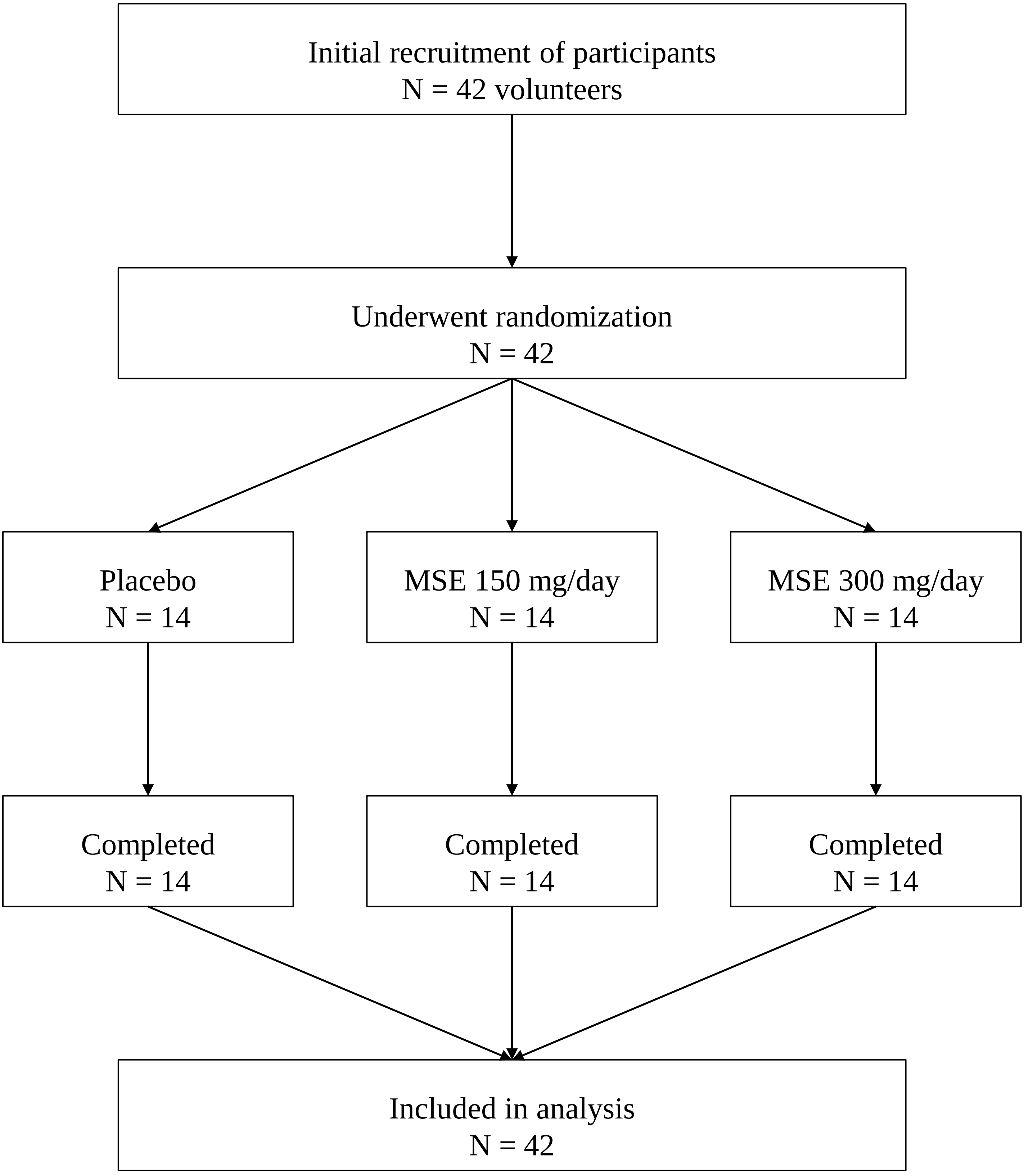

Supplementary Figure S2. Flowchart of participants throughout the study from recruitment to inclusion in analysis.
MSE, melinjo seed extract.

***ADIPOQ* rs3774261 A/A genotype carriers**

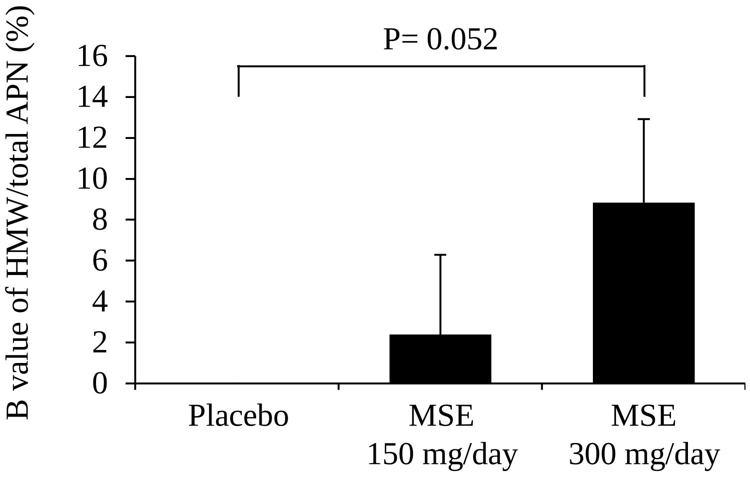

***ADIPOQ* rs182052 A/A genotype carriers**

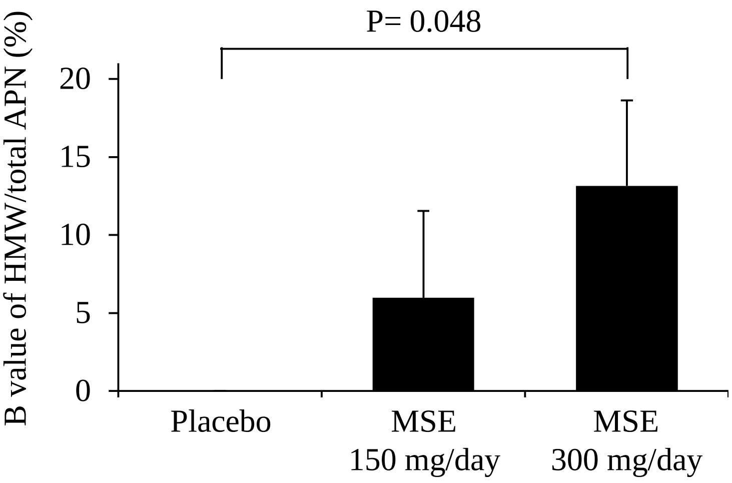

***ADIPOQ* rs3774261 A/G or G/G genotype carriers**

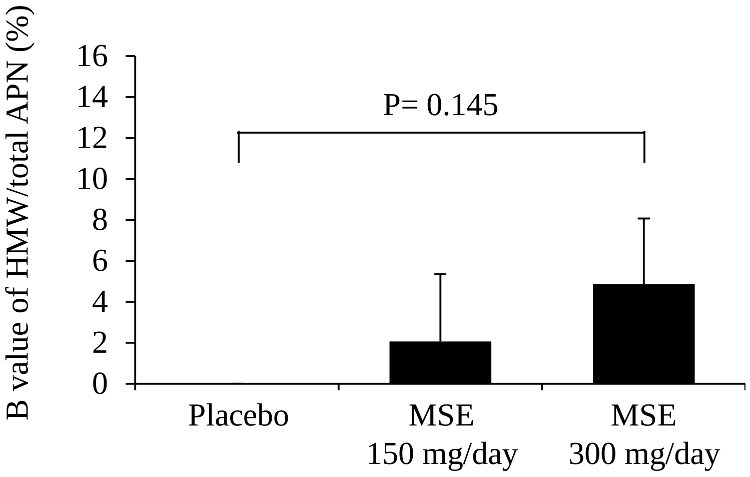

***ADIPOQ* rs182052 A/G genotype carriers**

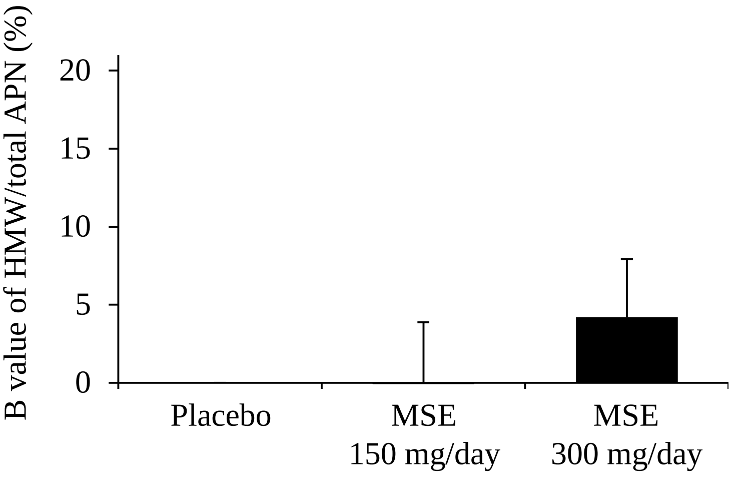

***ADIPOQ* rs182052 G/G genotype carriers**

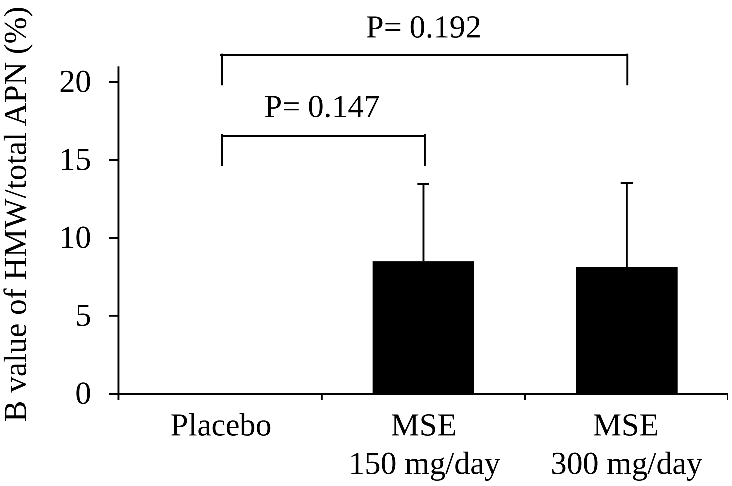

Supplementary Figure S3. The effects of the *ADIPOQ* rs3774261 and rs182052 genotypes and the administration of MSE on the ratio of HMW/total APN. The bars and error bars represent the unstandardized partial regression coefficients (B values) and SEs, respectively. B values were adjusted by the ratios of HMW/total APN before the administration of test tablets. ADIPOQ, adiponectin gene; MSE, melinjo seeds extract; HMW, high molecular weight; APN, adiponectin; SE, standard error.

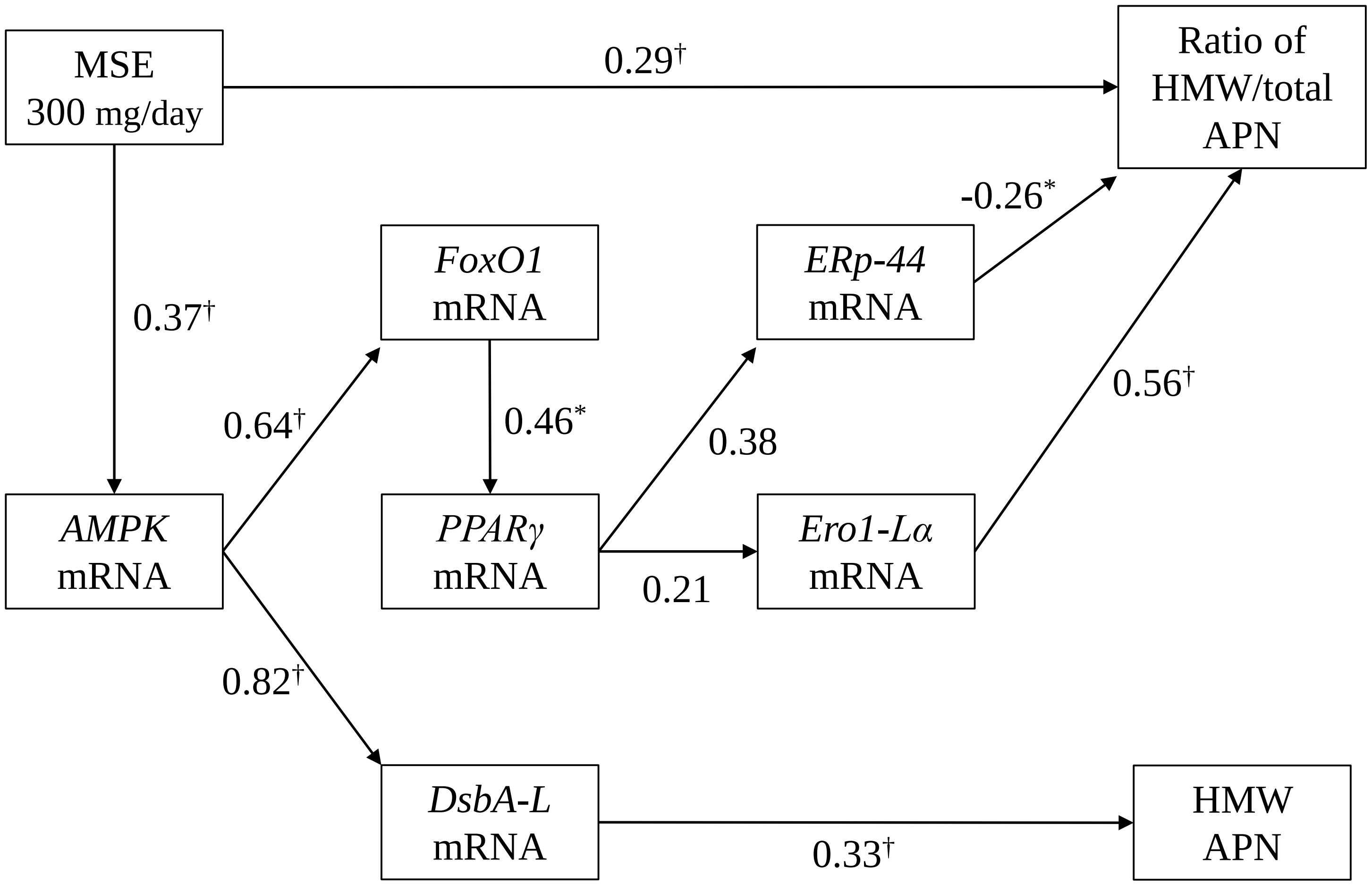

Supplementary Figure S4. The structural equation modeling diagram of MSE administration and APN multimerization in young, healthy volunteers with the *DsbA-L* rs1917760 T allele. Lines with numbers indicate significant paths with standardized partial regression coefficients (* $P < 0.05$, † $P < 0.01$). Arrows represent an association between two factors. The β values ranged from -1 to 1, with a positive value representing a positive correlation and negative value representing a negative correlation. The GFI, NFI, CFI and RMSEA of this model were 0.812, 0787, 0.903 and 0.157, respectively. MSE, melinjo seeds extract; APN, adiponectin; DsbA-L, disulfide-bond A oxidoreductase-like protein; AMPK, 5'-Adenosine monophosphate-activated protein kinase; FoxO1, forkhead box protein O1; PPARγ, peroxisome proliferator-activated receptor γ; ERp-44, endoplasmic reticulum protein 44; Ero1-Lα, endoplasmic reticulum oxidoreductase 1-like protein α; HMW, high-molecular-weight; GFI, goodness of fit index; NFI, Bentler-Bonett normed fit index; CFI, comparative fit index and RMSEA, root mean square error of approximation.

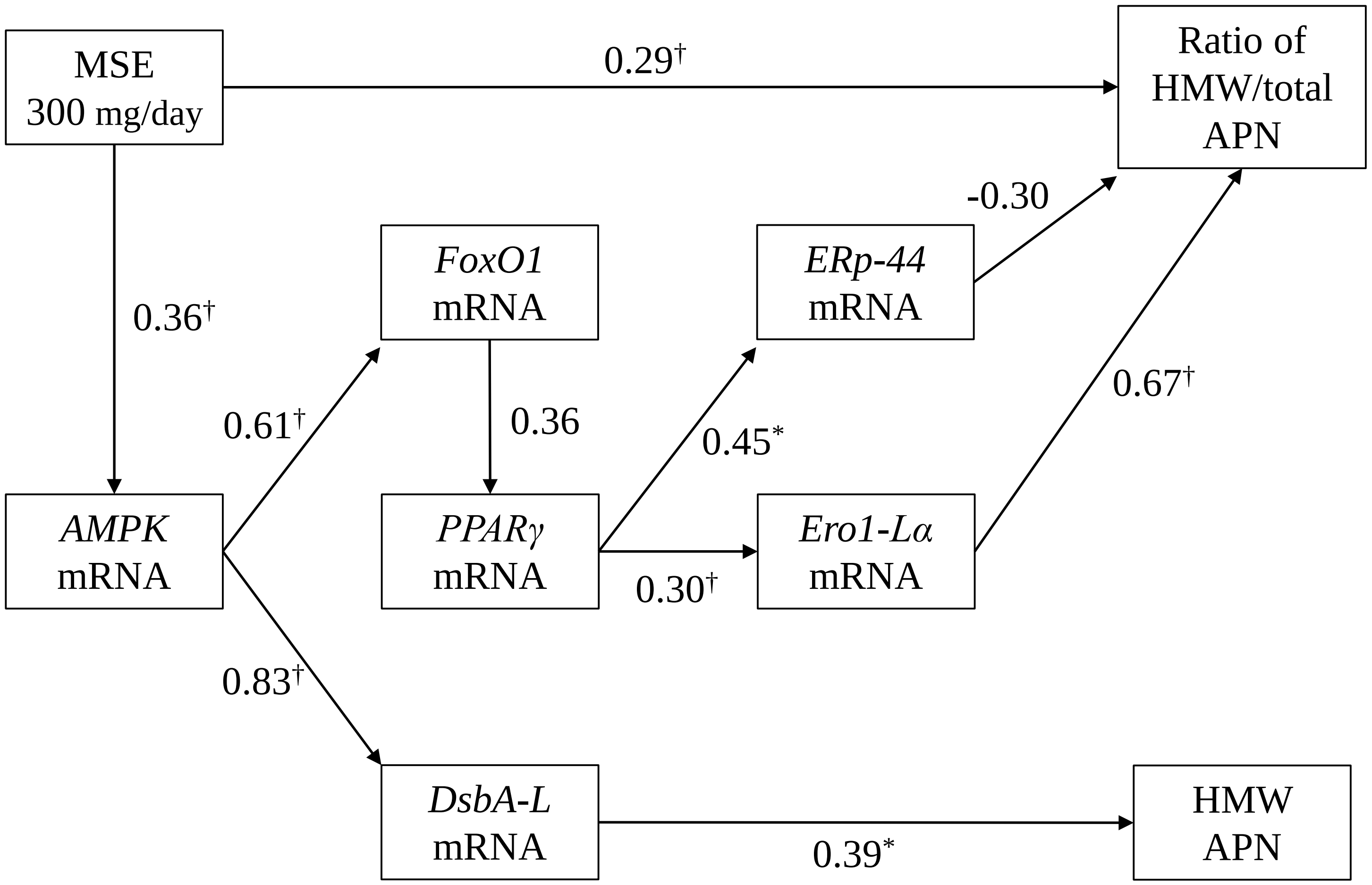

Supplementary Figure S5. The structural equation modeling diagram of MSE administration and APN multimerization in young, healthy volunteers with the *DsbA-L* G/T genotype. Lines with numbers indicate significant paths with standardized partial regression coefficients (* $P < 0.05$, † $P < 0.01$). Arrows represent an association between two factors. The β values ranged from -1 to 1, with a positive value representing a positive correlation and negative value representing a negative correlation. The GFI, NFI, CFI and RMSEA of this model were 0.757, 0688, 0.767 and 0.260, respectively. MSE, melinjo seeds extract; APN, adiponectin; DsbA-L, disulfide-bond A oxidoreductase-like protein; AMPK, 5'-Adenosine monophosphate-activated protein kinase; FoxO1, forkhead box protein O1; PPARγ, peroxisome proliferator-activated receptor γ; ERp-44, endoplasmic reticulum protein 44; Ero1-Lα, endoplasmic reticulum oxidoreductase 1-like protein α; HMW, high-molecular-weight; GFI, goodness of fit index; NFI, Bentler-Bonett normed fit index; CFI, comparative fit index and RMSEA, root mean square error of approximation.

A

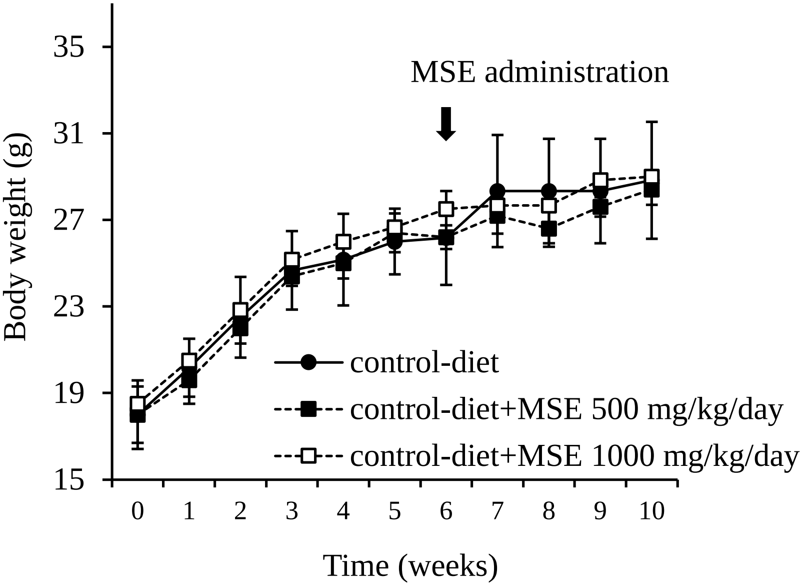

Supplementary Figure S6. The effects of MSE administration to control-diet-fed mice on the weight status, fasting blood glucose and fat mass. A: Body weight changes in control-diet-fed mice treated with vehicle or MSE (500 or 1000 mg/kg/day). **B** and **C**: Epididymal fat mass (**B**) and subcutaneous fat mass (**C**) levels in control-diet-fed mice treated with vehicle or MSE (500 or 1000 mg/kg/day). Data are means ± standard errors; n = 6 mice/group. P values were calculated by Dunnett's procedure (*P < 0.05, †P < 0.01). MSE, melinjo seeds extract; HFD, high-fat diet.

B

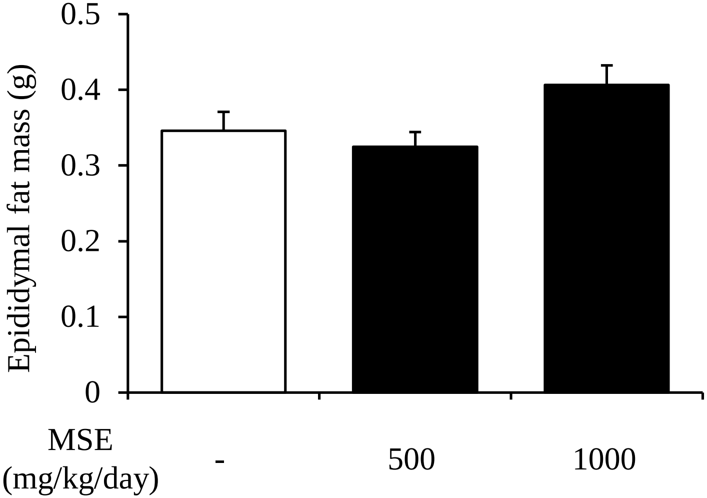

C

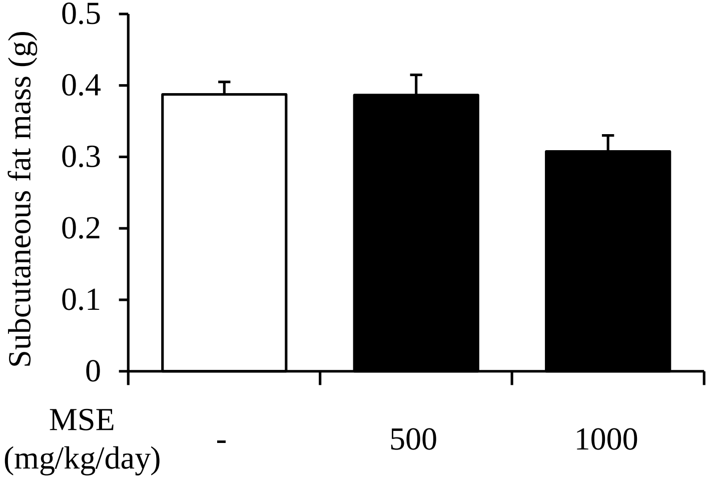

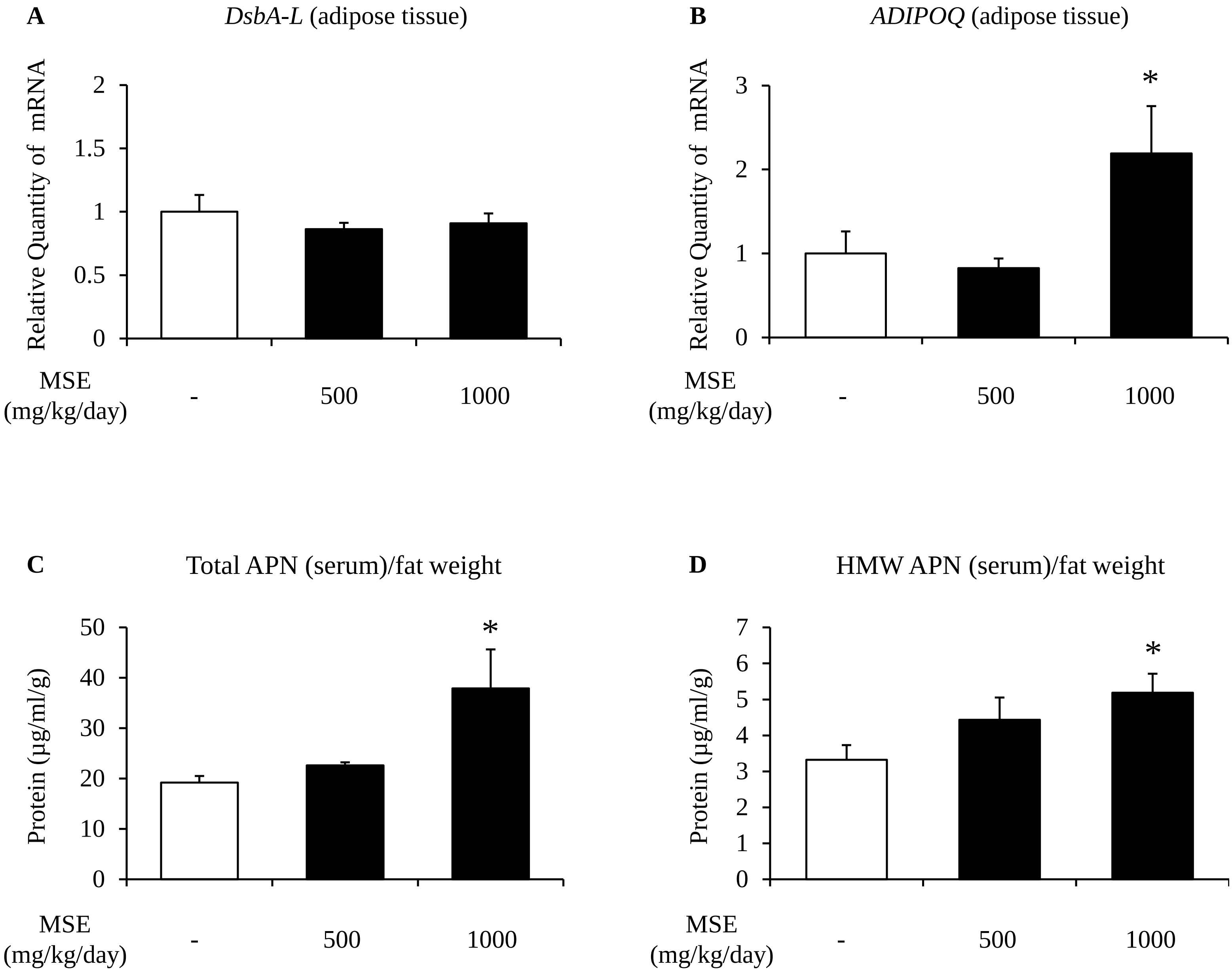

Supplementary Figure S7. The effects of MSE administration to control-diet-fed mice on the mRNA expression of *DsbA-L*, *ADIPOQ* and protein levels of APN. A: The mRNA expression of *DsbA-L* (adipose tissue) in control diet-fed mice treated with vehicle or MSE (500 or 1000 mg/kg/day). **B**: mRNA expression of *ADIPOQ* (adipose tissue) in control diet-fed mice treated with vehicle or MSE (500 or 1000 mg/kg/day). **C** and **D**: Serum adiponectin levels adjusted for the fat mass in control diet-fed mice treated with vehicle or MSE (500 or 1000 mg/kg/day). Data are means ± standard errors; n = 6 mice/group. P values were calculated by Dunnett procedure (*$P < 0.05$). MSE, melinjo seeds extract; DsbA-L, disulfide-bond A oxidoreductase-like protein; ADIPOQ, adiponectin gene; APN, adiponectin; HMW, high-molecular-weight.
